# RNA-binding proteins and exoribonucleases modulating miRNA in cancer: the enemy within

**DOI:** 10.1038/s12276-024-01224-z

**Published:** 2024-05-01

**Authors:** Yoona Seo, Jiho Rhim, Jong Heon Kim

**Affiliations:** 1 Cancer Molecular Biology Branch, Research Institute, National Cancer Center, Goyang, 10408 Korea; 2 Department of Cancer Biomedical Science, Graduate School of Cancer Science and Policy, National Cancer Center, Goyang, 10408 Korea

**Keywords:** miRNAs, Drug development

## Abstract

Recent progress in the investigation of microRNA (miRNA) biogenesis and the miRNA processing machinery has revealed previously unknown roles of posttranscriptional regulation in gene expression. The molecular mechanistic interplay between miRNAs and their regulatory factors, RNA-binding proteins (RBPs) and exoribonucleases, has been revealed to play a critical role in tumorigenesis. Moreover, recent studies have shown that the proliferation of hepatocellular carcinoma (HCC)-causing hepatitis C virus (HCV) is also characterized by close crosstalk of a multitude of host RBPs and exoribonucleases with miR-122 and its RNA genome, suggesting the importance of the mechanistic interplay among these factors during the proliferation of HCV. This review primarily aims to comprehensively describe the well-established roles and discuss the recently discovered understanding of miRNA regulators, RBPs and exoribonucleases, in relation to various cancers and the proliferation of a representative cancer-causing RNA virus, HCV. These have also opened the door to the emerging potential for treating cancers as well as HCV infection by targeting miRNAs or their respective cellular modulators.

## Exploring RBPs and exoribonucleases modulating miRNAs in cancer and HCV proliferation

For many years, scientists have been investigating the complex world of miRNAs. These tiny molecules can bind to target mRNAs, influencing translation and broadly affecting gene expression. This review delves into the intricate processes that modulate the biogenesis and degradation/trimming of miRNAs, with a focus on RNA-binding proteins (RBPs) and exoribonucleases. The review also underscores the importance of these miRNA modulators in the proliferation of the hepatitis C virus (HCV), which can chronically lead to liver cancer. It discusses how certain RBPs and exoribonucleases interplay with liver-specific miR-122 and HCV RNA, offering new paths for therapeutic strategies against HCV. In summary, this review illuminates key evidence that RBPs and exoribonucleases affect miRNAs in various cancers. This comprehensive understanding could lead to advancements in how RBPs and exoribonucleases can be targeted in the therapeutic strategies of cancers.

## Introduction

MicroRNAs (miRNAs), a class of small noncoding RNAs approximately 22 nucleotides in length, are known to play fundamental roles in the regulation of gene expression. A single miRNA can exhibit complementary base pairing with multiple potential sites of cellular mRNA transcripts, implying that miRNAs can affect the expression of a wide range of genes^1,2^. Thus, miRNAs play multifaceted roles in various cellular processes ranging from differentiation to disease progression. The biogenesis and turnover of miRNAs are tightly regulated at multiple levels of maturation or degradation by various RNA-binding proteins (RBPs) and ribonucleases^3^. In addition to conventional endoribonucleases such as Drosha, Dicer, and AGO2, there may be more unconventional elements beyond the basic factors involved in miRNA modulation.

Dysregulation of miRNAs is a common event in tumor progression through the inhibition of important tumor suppressors or the increased expression of oncogenic factors^4^. Similarly, alterations in or dysregulation of RBPs and exoribonucleases, which modulate these miRNAs, have been explored for their roles in tumorigenesis. In addition to their roles in conventional nonviral tumorigenesis, several RBPs and exoribonucleases have been found to affect hepatitis C virus (HCV) proliferation, which can, in the presence of chronic infection, lead to hepatocellular carcinoma (HCC) by regulating the liver-specific miRNA miR-122^5,6^. In light of these findings, in this review, HCV is considered as a representative oncogenic virus that mediates miRNA regulation. Evidence regarding the dysregulation of miRNA biogenesis has been gradually increased through various cancer-related studies; RBPs and exoribonucleases have been shown to be involved in miRNA turnover via direct or indirect mechanisms^7,8^. In addition, accumulating research has demonstrated that many RBPs can modulate microprocessors and directly bind to primary (pri-) and/or precursor (pre-)miRNAs to promote their maturation^3^.

RBPs are a group of functional proteins with an RNA-binding domain (RBD) through which they can recognize particular sequences on and bind to specific target RNAs, eventually affecting RNA stability and consequent function^9^. Although many studies have focused on the regulation of RBP expression by miRNA targeting, RBPs are also essential cellular factors for modulating miRNA expression through controlling miRNA biogenesis and turnover^3^.

For instance, heterogeneous nuclear ribonucleoproteins (hnRNPs) are well-known RBPs because of their roles in alternative splicing, mRNA stability, and translation^10,11^. Notably, hnRNPs have also been found to directly or indirectly influence miRNA expression by modulating pri- and/or pre-miRNA processing^12^. Furthermore, hnRNPs can indirectly compete with or inhibit the miRNA-induced silencing complex (miRISC). In addition, ELAVL1/HuR, a well-known posttranscriptional modulator, is an RBP known to facilitate the expression of viral proteins and play a role in productive viral infections^13^. Although ELAVL1/HuR is known to be closely associated with HCC-causing HCV, it was recently found to significantly enhance HCV RNA function by protecting the HCV replication-augmenting miRNA miR-122 from pervasive host exoribonucleases^14^. Thus, in virus-related tumorigenesis, such as that leading to HCV-associated HCC, RBPs play an important role in miRNA homeostasis and disease progression. In this review, in-depth investigations of representative critical RBPs are discussed to highlight the extent of the importance of such emerging miRNA regulators.

Furthermore, exoribonucleases have been demonstrated to play an important role in miRNA degradation as well as in the maintenance of miRNA stability, indicating that they are critical for miRNA biogenesis and turnover. Recently, there has been increasing evidence that they may play a greater role in cancer progression than previously appreciated. For instance, XRN1, a 5′–3′ exoribonuclease, was found to affect the turnover of several tumor-suppressive miRNAs beyond the conventional miRNA degradation mechanism by forming complexes with other factors such as IFIT5^15^. Another 5′–3′ exoribonuclease, XRN2, was also found to be involved not only in miRNA decay but also in miRNA maturation through binding to pre-miRNAs and was shown to be linked to the promotion of epithelial–mesenchymal transition (EMT) in cancer^16^. In addition, both XRN1 and XRN2 can attack the HCV RNA genome and compete with HCV-protective miR-122 during HCV replication, indicating that they play roles in maintaining cellular homeostasis and acting as a primary host defense system^17,18^. Recently, poly(A)-specific ribonuclease (PARN), a representative 3′–5′ exoribonuclease known to be a key modulator of mRNAs and small RNAs, was shown to degrade miR-7 during the development of glioblastoma malignancy^19^.

More related studies would be helpful for elucidating the mechanisms underlying miRNA biogenesis and turnover. Moreover, drug development and novel therapeutic strategies could be adapted accordingly for the treatment of rare and intractable cancers.

## Roles of miRNAs in tumorigenesis

Recent progress in miRNA research has revealed that some miRNAs play important roles in human diseases such as cancer. Since miRNAs can regulate more than 30% of human genes, they have been extensively studied and demonstrated to be involved in a broad range of events in cancer biology, from cancer initiation to tumor progression and metastasis^4^. Aberrant expression of miRNAs is commonly observed in various cancer types and has been revealed to be associated with diverse cellular environments in cancers. Accumulating evidence has demonstrated that numerous miRNAs can behave as tumor suppressors or oncogenic factors. In this regard, miRNAs have been considered as diagnostic markers and potential therapeutic targets for various cancers^20,21^.

Moreover, miRNAs have been shown to be involved in virus-mediated tumorigenesis^5^. It has been consistently reported that seven types of viruses contribute to the development of specific cancers in humans, accounting for ~13‒15% of all human cancer cases globally^5,22^. These oncogenic viruses include four DNA viruses [Epstein‒Barr virus (EBV), hepatitis B virus (HBV), human herpesvirus 8 (HHV-8), and human papillomavirus (HPV)] and three RNA viruses [hepatitis C virus (HCV), human immunodeficiency virus (HIV), and human T-lymphotrophic virus type 1 (HTLV-1)]^5^.

These oncogenic viruses have been shown to control and utilize intracellular components such as miRNAs and regulate gene expression in the host and viral genomes^5^. In addition to the two retroviruses (HIV and HTLV-1) that use their own systems to transcribe DNA from RNA and then incorporate this DNA into the host genome, HCV is the only positive-sense single-stranded RNA virus that is released into the cytoplasm of host cells for replication, which, in the presence of chronic infection, leads to HCC^5,6^. Since HCV RNA has been shown to be mutually associated with the liver-specific miR-122 and other factors related to cellular function, HCV is dealt with as a representative oncogenic virus mediating miRNA regulation in this review.

## Close encounters between miRNAs and their cellular modulators—RBPs and exoribonucleases

In recent studies, posttranscriptional modifications of miRNAs by host cellular modulators such as RBPs and exoribonucleases have been extensively investigated for their significance in cancer progression^23,24^. The effects of RBPs and exoribonucleases on miRNA regulation are conflicting and not always consistent for all types of miRNAs and cancers. However, it is apparent that RBPs and exoribonucleases can modulate miRNA expression and/or function by regulating miRNA biogenesis and turnover. Thus, miRNA dysregulation in cancer can be attributed to regulatory abnormalities arising via alteration in RBPs and exoribonucleases. In this respect, this review comprehensively summarizes and discusses the noteworthy cellular modulators—RBPs and exoribonucleases—that are implicated in posttranscriptional regulation of miRNAs in cancer. Furthermore, this review is helpful for establishing a practical rationale for the development of cancer-related miRNA modulators as promising cancer therapeutic agents or treatment strategies.

## RBPs modulating miRNAs in cancer: conventional RBPs

RBPs are able to bind specific target RNAs through conventional RBDs, noncanonical enzymatic cores, and adapted intrinsically disordered regions^9^. RBPs and miRNAs generally interact via conventional mechanisms, which affects miRNA biogenesis and/or functions, including processing of pri- and pre-miRNA and RNA-induced silencing complex (RISC) loading. These interactions can result in either cooperative, positive regulation or competitive, negative regulation depending on the cellular context^25^. Considering the functions associated with miRNAs, various RBPs have been found to affect miRNA processing in both transcriptional and posttranscriptional regulation, leading to cancer progression (Table 1). Simultaneously, alterations in RBP expression or activity attributed to cancer progression could result in aberrant miRNA processing from biogenesis to turnover. Although the dysregulated RBPs targeted by miRNAs during cancer progression are relatively well investigated, the roles of RBPs that modulate miRNAs in cancer have been insufficiently reviewed yet.

**Table 1:** Summary of representative RNA-binding proteins (RBPs) modulating miRNAs associated with various cancers.

RBP	Target miRNAs associated with cancer	Mechanism of miRNA modulation	Functions associated with miRNA modulation	Ref.
hnRNP A1	miR-18a; tumor-suppressive in ovarian cancer^30^	Pri-miRNA processing	Directly binds to pri-miR-18a by recognizing the terminal loop; Promotes Drosha processing and miRNA maturation by inducing a change to a relaxed conformation at the Drosha cleavage sites	^12,28,29^
	let-7a-1; representative tumor-suppressive miRNA in various cancers	Pri-miRNA processing	Competes and interferes with KSRP for pri-let-7a-1 binding by interacting with the terminal loop of pri-let-7a-1 and blocking its Drosha-mediated processing	^32^
hnRNP A2/B1	miR-506; lung cancer	mRNA targeting	Promotes miR-506-mediated CDK6 silencing by modulating the 3′UTR stem structure of CDK6 mRNA and facilitating AGO2 binding	^34^
hnRNP D	miR-122; HCC	Pre-miRNA processing	Indirectly blocks the processing of pre-miR-122 into mature miR-122 by suppressing Dicer expression via interacting with the 3′UTR and coding region of Dicer mRNA	^35,36^
hnRNP I	let-7, miR-21; representative tumor-suppressive and oncogenic miRNAs in various cancers, respectively	mRNA targeting	Affects the association between miRNAs and their target mRNAs by interacting with miRNAs and AGO2 via the miRISC	^39^
	miR-101; lung cancer		Facilitates AGO2 association with MCL1 mRNA via the miR-101-loaded miRISC	^40^
hnRNP Q	let-7a	Pri-miRNA processing	Promotes Drosha processing of let-7 via direct binding to the pri-let-7a by recognizing the terminal loop and associating with DGCR8	^44^
hnRNP L	miR-297, miR-299; AML	mRNA targeting	Competes with miR-297 and miR-299 for binding to the CA-rich element in the VEGFA 3′UTR under hypoxic conditions	^46^
Lin28A	let-7; breast cancer (let-7a)^65^, lung cancer (let-7g)^66^, lung cancer(let-7a)^67^	Pre-miRNA processing	Binds to pre-let-7 by recognizing the terminal loop and inhibits Dicer processing in the cytoplasm along with recruiting TUT4, which elongates the 3′-end with an oligomeric (U) stretch	^56–59^
Lin28B	let-7; HCC (let-7/miR-98)^68,69^, colon cancer (let-7a/b)^70^	Pri-miRNA processing	Binds to pri-let-7 by recognizing the terminal loop and inhibits Drosha-mediated processing in the nucleus in a TUT4-independent manner	^54,60^
ELAVL1/HuR	miR-494; cervical cancer	mRNA targeting	Competes with miR-494 to promote the translation of nucleolin mRNA by binding to the 3′UTR of the mRNA and protecting it from interacting with the miR-494-loaded miRISC	^78^
	miR-300; gastric cancer	mRNA targeting	Disrupts the miR-300 association with UBE2C mRNA by competing for binding to the overlapping binding sites in the UBE2C 3′UTR	^79^
	miR-122; HCC	mRNA targeting	Competes with miR-122 for CAT-1 mRNA by binding to the 3′UTR of the mRNA	^76^
	let-7; cervical cancer	mRNA targeting	Facilitates the interaction of the let-7-loaded miRISC with the 3′UTR of c-Myc mRNA by recognizing its binding motifs	^81^
	miR-19b; breast cancer	mRNA targeting	Facilitates the interaction of the miR-19b-loaded miRISC with the 3′UTR of ABCB1 mRNA by binding to ELAVL1/HuR, which contains overlapping binding sites with miR-19b in ABCB1 mRNA	^82^
KSRP	let-7a-1	Pri-miRNA processing	Competes with hnRNP A1 for binding to the terminal loop of pri-let-7a-1 and enhances its Drosha-mediated processing	^32,86^
	miR-23a; NSCLC	^a^N.D.	Negatively regulates oncogenic EGR3 mRNA stability through promoting the maturation of miR-23a targeting EGR3 mRNA	^87^
	miR-192-5p; breast cancer (mammary gland)	N.D.	Indirectly abrogates the metastatic potential by enhancing the expression of miR-192-5p, which can target EMT-related factors	^88^
	miR-26a; SCLC	N.D.	Indirectly inhibits tumor suppressor PTEN mRNA expression by augmenting the maturation of miR-26a, which can target the PTEN mRNA 3′UTR	^89^
	miR-21, miR-130b, miR-301a; esophageal squamous cell carcinoma	N.D.	Increases the expression of oncogenic miRNAs and represses their target mRNAs, which are known to inhibit EMT in cancer	^91^
	miR-629-5p; ccRCC (clear cell renal cell carcinoma)	N.D.	Either directly or indirectly attenuates NEDD4L mRNA stability by binding to the ARE within the mRNA 3′UTR and promoting the maturation of miR-629-5p, which targets NEDD4L mRNA	^92^
TRIM71/LIN41	let-7, miR-124	mRNA targeting	Potentially antagonizes AGO2 action by mediating the ubiquitination of AGO2 and interferes with miRNA (let-7, miR-124)-mediated gene silencing	^96^
	let-7	Pre-miRNA processing (post-Drosha step)	Indirectly promotes let-7 maturation by attenuating Lin28B protein stability via catalyzing its polyubiquitination through interaction with the C-terminal unique amino acid stretch of Lin28B	^94,95^
	let-7	Pre-miRNA degradation	Promotes the degradation of pre-let-7 via a direct interaction with Lin28A/TUT4, thus disturbing let-7 maturation and indirectly stabilizing let-7 target mRNAs	^97^
	let-7	mRNA targeting	Suppresses the activity of mature let-7 miRNA via an RNA-dependent interaction with AGO2	^97^
ARS2	miR-21	Pri-miRNA processing	Mediates the interaction between the CBC complex and the Drosha-DGCR8 heterodimeric pri-miRNA processing machinery	^105^
	miR-6798-3p; glioblastoma	N.D.	Facilitates cancer progression by inhibiting p53/p21-mediated apoptosis through upregulation of miR-6798-3p	^111^
TARBP	miR-17, miR-20a, miR-92a (proto-oncomiRs)^121–123^ let-7	Pre-miRNA processing	Phosphorylated TARBP increases the stabilization and expression of the miRNA processing complex, leading to global miRNA profile changes	^120^
	miR-21	Pre-miRNA processing	SUMOylated TARBP2 strengthens miRNA-induced gene silencing, not miRNA production, by forming more of functional miRISC via increasing its binding affinity for pre-miRNA and stabilizing AGO2	^124^
	miR-145; HCC	Pre-miRNA processing (not validated.)	TARBP2 facilitates miR-145 maturation, leading to repression of SERPINE1 mRNA, which is targeted by miR-145	^128^
	Overall miRNAs; CRC	Pre-miRNA processing	Activated TARBP2 can globally change miRNA profiles by promoting pre-miRNA processing	^130^
ADAR1	miR-302/367 cluster; gastric cancer	Pri-miRNA processing	Promotes Drosha processing of the pri-miR-302/367 cluster	^141^
	miR-125a; chordoma let-7; leukemia	Pri-miRNA processing	Impairs the biogenesis of tumor-suppressive miRNAs (miR-125a, let-7) at the Drosha cleavage step	^140,143^
	miR-21, miR-18a, miR-210, miR-155, miR-181a, miR-19a; OSCC	Pre-miRNA processing	Facilitates the biogenesis of several oncogenic miRNAs by interacting with Dicer in the cytoplasm	^142^
	miR-10a; chordoma	Pre-miRNA processing	Disturbs the biogenesis of tumor-suppressive miR-10a at the Dicer processing step	^143^
	miR-455; melanoma	Pri-miRNA processing	Attenuates the Drosha binding capacity by interacting and editing pri-miR-455	^146^
	Overall miRNAs; melanoma	Pri-miRNA processing	Alters miRNA expression profiles by impeding generation of the Drosha-DGCR8 complex through the formation of a heterodimeric complex with DGCR8	^149^
	let-7, miR-103-3p, miR-181a; cervical cancer	Pre-miRNA processing	Facilitates Dicer-mediated cleavage of pre-miRNAs and increases miRISC-induced mRNA silencing by interacting with Dicer to form a heterodimeric complex	^150^
ADAR2	miR-21, miR-221/222; glioblastoma	Pri-miRNA processing	Reduces the expression levels of oncogenic miRNAs (miR-21, miR-221/222) by interfering with Drosha processing through editing of the stem‒loop structure	^147^
	miR-376a-3p; glioblastoma	Pri-miRNA processing	Contributes to the accumulation of normally edited miR-376a-3p through modification of pri-miR-376a1	^148^
	miR-214; HCC	Pri-miRNA processing	Disturbs the processing of pri-miR-214 into mature miR-214 by increasing the number of mismatches between pri-miR-214 and its antisense RNA transcripts through modifying the nucleotides of the transcripts	^139^
DDX3	miR-1, miR-141, miR-145, miR-19b, miR-20a, miR-34a; various cancers	Pri-miRNA processing	Changes the expression profiles of a subset of miRNAs by facilitating pri-miRNA processing and maturation through binding to the Drosha-DGCR8 microprocessor and increasing its processing activity	^159^
	miR-20a; leukemia	Pri-miRNA processing	Facilitates the biogenesis of miR-20a by increasing its stability through binding to pri-miR-20a	^160^
	miR-200b, miR-200c, miR-122, miR-145; HCC	Pri-miRNA processing	Facilitates the transcription of tumor-suppressive miRNAs by enhancing the interaction of transcription factors and interfering with DNMT3A binding and hypermethylation on promoter regions of the miRNAs	^161^
DDX5	miR-34a, miR-182, miR-196-a2; breast cancer	Pri-miRNA processing	Enables LMTK3 (Lemur tyrosine kinase 3) to facilitate the maturation of pri-miRNAs (pri-miR-34a, pri-miR-182, and pri-miR-196-a2)	^165^
	miR-21; breast cancer	Pri-miRNA processing	Involved in SMAD signaling-induced miR-21 maturation by generating a protein complex with Drosha-DGCR8	^166,167^
	miR-10b; breast cancer	Pri-miRNA processing	PAK5 (p21-activated kinase 5)-mediated phosphorylation and subsequent SUMOylation increase the biogenesis of oncogenic miR-10b through augmenting the interaction of DDX5 with the Drosha-DGCR8 complex	^168^
	let-7	Pre-miRNA processing (duplex structure)	Promotes the formation of the let-7-loaded miRISC by unwinding the pre-let-7 duplex structure via its helicase activity	^169^
DDX6	let-7; cervical cancer	mRNA targeting	Promotes let-7-mediated gene silencing by directly interacting with AGO1 and AGO2, associating with the RISC to form the let-7-mediated miRISC	^175^
DDX17	miR-26a2; neuroblastoma	Pri-miRNA processing	Regulates nuclear maturation of pri-miR-26a2 during early stages of neuronal differentiation	^179^
	Various miRNAs (miR-125b-1, miR-21, let-7g, etc.)	Pri-miRNA processing	Influences the pri-miRNA processing activity of the Drosha-DGCR8 microprocessor complex along with the YAP protein in a cell density-dependent manner by recognizing a sequence motif in the 3′-flanking segment of pri-miRNAs	^180^
	miR-149-3p; CRC	N.D.	Inhibits the expression of tumor-suppressive miR-149-3p, resulting in increased expression of its target CYBRD1	^181^
	miR-125a	Pri-miRNA processing	Recognizes specific sequence motifs in pri-miRNAs via the RMFQ (R250, M254, F256, and Q259) groove in its catalytic core and promotes the processing of pri-miRNAs by remodeling the 3′ flanking region	^183^
DDX23	miR-21; glioblastoma	Pri-miRNA processing	Posttranscriptionally facilitates pri-miR-21 processing by interacting with the Drosha-DGCR8 microprocessor complex via its intrinsic helicase activity	^188^

^a^N.D.; not described.

## HnRNP family

**Fig. 1:**
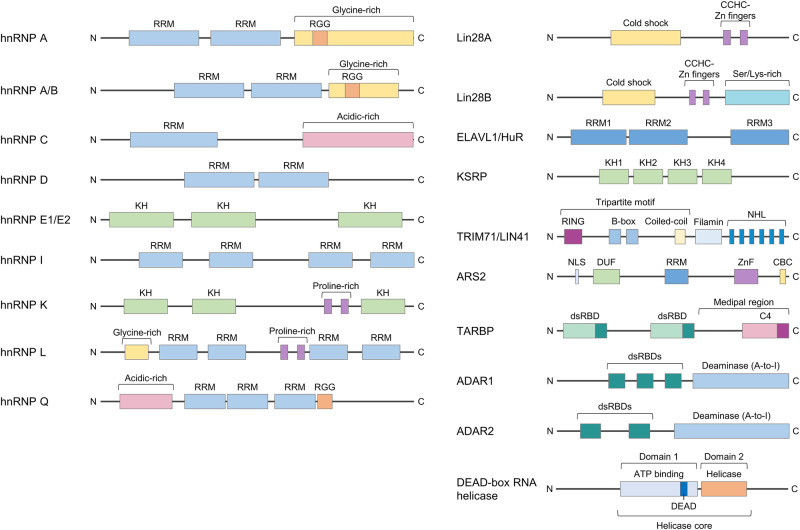
Schematic diagram of the structure of miRNA-modulating RBPs with functional domains and motifs. The hnRNP family (left). hnRNPs have different molecular weights ranging from 34 to 120-kDa, and approximately 20 major hnRNPs, from Al to U, have been characterized in humans. Among the hnRNP family, several representatives known to be involved in miRNA regulation were selected for presentation. Three unique RNA-binding domains (RBDs) in hnRNP family members are required for RNA binding. RRM, RNA recognition motif; KH, K-homology domain; and RGG, RNA-binding domain consisting of Arg-Gly-Gly repeats. Various miRNA-modulating RBPs with functional domains and motifs (right). Lin28A contains a cold-shock domain (CSD) and CCHC-type Zn finger motifs for nucleic acid binding. Lin28B also contains a CSD, CCHC-type Zn finger motifs for nucleic acid binding, and a C-terminal Ser/Lys-rich motif. ELAVL1/HuR contains three RRMs (RRM1–3) for RNA recognition. KSRP contains four KH domains (KH1–4) for RNA recognition. TRIM71/LIN41 contains a RING (Really Interesting New Gene) domain for E3 ubiquitin ligase activity, two B-boxes, a coiled-coil domain, a filamin domain, and a unique C-terminal NHL (NCL-1, HT2A2, and LIN41)-repeat motif for RNA binding. ARS2 contains an N-terminal nuclear localization signal (NLS), a domain of unknown function (DUF), a central RRM motif for RNA substrate binding, a zinc finger (ZnF), and a C-terminal proline-rich unique nuclear cap-binding complex (CBC)-binding motif. TARBP contains two double-stranded RNA binding domains (dsRBDs), and C4 in the Medipal region is required for its interaction with Dicer. ADAR1 and ADAR2 contain dsRBDs and an adenosine (A)-to-inosine (I)-deamination catalytic domain. DEAD-box RNA helicases contain a common DEAD (Asp-Glu-Ala-Asp) motif for ATP-binding and a helicase domain. A detailed description of the RBPs, with their functional domains and motifs is provided in the text.

HnRNPs are a large group of RBPs that are generally involved in various processes in RNA metabolism, such as transcription, translation, stabilization, and splicing^10,11^. To date, at least 20 hnRNPs have been characterized in humans (A1 to U), and they exhibit some common structural features with different RBDs and functional properties^10,11,26^. There are four types of RBDs in hnRNPs: the RNA recognition motif (RRM), the quasi-RRM (qRRM), the RGG box in the glycine-rich domain, and the K-homology (KH) domain (Fig. 1)^11^. It has been reported that hnRNPs are mostly located in the nucleus under steady-state conditions; however, they can be localized in diverse compartments in cells with distinct sequence binding affinities depending on their specific structures and intrinsic functions^10,26,27^. It has also been found that the localization and diversity of RBDs lead to the multifarious functions of hnRNPs^11^. Based on these characteristics, hnRNPs have been widely investigated for their ability to coordinate various cellular events in human diseases, including cancers^11^. In addition, distinct expression of hnRNPs has been observed in different tissues, and dysregulated hnRNP expression has been detected in numerous cancers, indicating that hnRNPs can play multiple roles in the progression of diverse cancers^11^. The accumulated evidence also indicates that hnRNPs can be involved in modulating miRNAs during tumorigenesis. In this regard, this section delineates several representative reports suggesting the roles of hnRNPs associated with miRNA regulation in cancer.

**Fig. 2:**
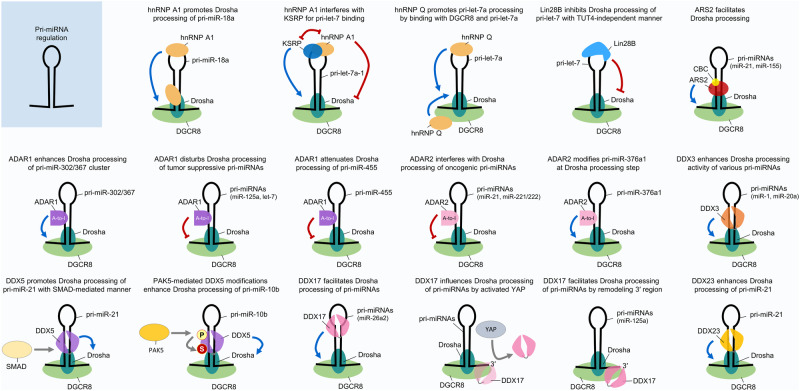
RBP action and interplay in pri-miRNA regulation. The figure illustrates several examples of the mechanism of action of RBP-mediated pri-miRNA regulation in cancer. RBPs have posttranscriptional mechanistic interactions with various pri-miRNA transcripts and the Drosha-DGCR8 microprocessor complex. Consequently, RBP-triggered miRNA-dependent signaling pathways affect tumor progression. A detailed description of the mode of action is provided in the text.

Accumulating studies have demonstrated that hnRNPs can directly or indirectly influence miRNA expression and activity by modulating miRNA biogenesis-related functional proteins such as microprocessor Drosha, Dicer, and AGO2. For instance, hnRNP A1 has been reported to participate in miRNA biogenesis, especially in pri-miRNA processing. It directly binds to pri-miR-18a by recognizing the terminal loop, promotes further Drosha processing and subsequent miRNA maturation by inducing a relaxed conformational change at Drosha cleavage sites (Fig. 2)^12,28,29^. The normally processed mature miRNA miR-18a-3p acts as a tumor suppressor by targeting the K-Ras oncogene in ovarian cancer^30^. Similarly, hnRNP A1 can also directly bind to the 5′-ends of miR-17 and miR-18 in conjunction with hnRNP C in thyroid cancer cells, thereby upregulating the expression of those miRNAs and promoting tumorigenic properties^31^.

However, the miRNA-modulatory functions of hnRNP A1 can exert both positive and negative effects on miRNA processing depending on its interaction with other functional partners. In contrast with the aforementioned evidence, another report showed that hnRNP A1 binds to the terminal loop of pri-let-7a-1 and blocks its Drosha-mediated processing; hnRNP A1 subsequently competes and interferes with a miRNA precursor maturation-promoting factor KSRP for binding to pri-let-7a-1 (Fig. 2)^32,33^. Eventually, hnRNP A1 can inhibit the biogenesis of representative tumor-suppressive miRNA, let-7a. This finding suggests that hnRNP A1 can affect cancer progression in various manners via complex regulatory mechanisms in miRNA regulation.

Furthermore, hnRNP A2/B1 was also reported to promote miR-506-mediated CDK6 silencing by modulating the 3′UTR stem structure of CDK6 mRNA to facilitate AGO2 binding in lung cancer cells^34^. Downregulation of CDK6 expression via miR-506 targeting induces G1 arrest; thus, hnRNP A2/B1 can eventually inhibit the proliferation of lung cancer cells.

HnRNP D, also known as AUF1, indirectly regulates miR-122 maturation in HCC^35^. It interacts with the 3′UTR and coding region of Dicer mRNA and suppresses Dicer expression in HCC^35,36^. As a result of Dicer inhibition by hnRNP D, the processing of pre-miR-122 to mature miR-122 is blocked in HCC^35^. The miR-122 was reported to be highly expressed in normal liver cells and is considered a tumor-suppressive miRNA in HCC^37^. Therefore, the actions of hnRNP D may lead to HCC progression.

HnRNP E1, also known as poly(rC)-binding protein (PCBP1)^10^, was reported to bind with AGO2 to stabilize miR-1, miR-133, and miR-206 in muscle cells (Fig. 4)^38^. Although this result was not obtained in cancer cells, it may suggest potential cancer-related roles for hnRNP E1 in regulating miRNAs during tumorigenesis.

HnRNP I, also known as PTB or PTBP1 (polypyrimidine track-binding protein 1), was reported to interact with various miRNAs and AGO2 via the miRISC, therefore affecting the interaction between the miRNA and its target mRNA (Fig. 4)^39^. Interestingly, the authors validated the results with both let-7 and miR-21, which are representative tumor-suppressive and oncogenic miRNAs, respectively. Considering this point, hnRNP I seems to diversely affect miRNAs overall regardless of their specific effect on cancers. Similarly, other research has demonstrated that hnRNP I can facilitate the association of AGO2 with MCL1 mRNA via the miR-101-loaded miRISC, thus inhibiting the expression of MCL1 and suppressing its antiapoptotic and prosurvival effects on lung cancer cells^40^. Through this evidence on hnRNP I, the comprehensive functions of hnRNP I should be separately verified in each cancer type to support its use as a potential anticancer therapeutic target.

HnRNP Q, also known as NS1-associated protein 1 (NSAP1) or synaptotagmin-binding cytoplasmic RNA-interacting protein (SYNCRIP), was shown to be involved in several pathways related to RNA metabolism, such as RNA maturation, editing, translational turnover, and HCV translation/replication^41–43^. Chen and colleagues demonstrated that hnRNP Q can regulate the expression of a subset of miRNAs, especially let-7a. It directly binds to pri-let-7a by recognizing the terminal loop of pri-let-7a and associates with DGCR8, thereby promoting processing via the Drosha-mediated microprocessor complex for miRNA maturation (Fig. 2)^44^.

In addition to the above-described miRNA-regulatory mechanisms of hnRNPs, the other mentioned manner also seems to be utilized according to the type of hnRNP. HnRNP Q was reported to have discriminatory ability for exosomal miRNAs via direct associations with specific extra-seed sequence motifs of miRNAs^45^. Because exosomal miRNAs have been shown to be critical in tumorigenesis, hnRNP Q may function in cancer progression by controlling the expression of exosomal miRNAs. In addition, HnRNP L was found to compete with miR-297 and miR-299, which bind to the CA-rich element (CARE) in the VEGFA 3′UTR, under hypoxic conditions (Fig. 4)^46^. While normally localized in the nucleus, hnRNP L can be translocated into the cytoplasm under hypoxic conditions. Then, hnRNP L can bind to VEGFA in the cytoplasm and inhibit the cellular activity of CARE-binding miRNAs (Fig. 4)^46^. Although this finding was validated in an acute myeloid leukemia (AML) cell line, hypoxia induction, the key physiological cue in these studies, is known to promote cancer progression in general. It would be intriguing to further investigate the role of hnRNP L in competing with miRNAs under hypoxic conditions in hypoxia-related tumorigenic environments in the near future.

Collectively, these diverse findings on several hnRNPs demonstrate their potential for clinical use as therapeutic targets in human diseases, especially cancers. These reports indicate that hnRNPs can modulate miRNAs in various ways by directly and indirectly controlling the biogenesis and turnover of miRNAs. Thus, specific regulation of the expression and activity of hnRNPs may be a promising treatment strategy for cancers.

## Lin28: Lin28A and Lin28B

The highly conserved RBP Lin28 contains two types of RBDs: a cold-shock domain (CSD) and a zinc finger domain (CCHC-ZnF) (Fig. 1)^47,48^. It was initially identified in *Caenorhabditis elegans* (*C. elegans*)^48^, and the mammalian genome encodes two paralogs, Lin28A and Lin28B, which are differentially localized and either synergistically or independently affect biological events depending upon the cellular context^47,49^. Lin28 has been demonstrated to be involved in regulating the pluripotency of embryonic stem cells and human somatic cells^50^. Subsequent studies revealed that Lin28 plays key roles in various human cancers, which are characterized by advanced histological grade, high clinical stage, poor differentiation, and high aggressiveness^51,52^. It is expressed predominantly in stem-like cells, not in normal cells, and it is abnormally activated in cancer cells^53^. The role and mechanism of Lin28 in cancer have been elucidated mainly through the interaction between the Lin28 and let-7 miRNAs.

**Fig. 3:**
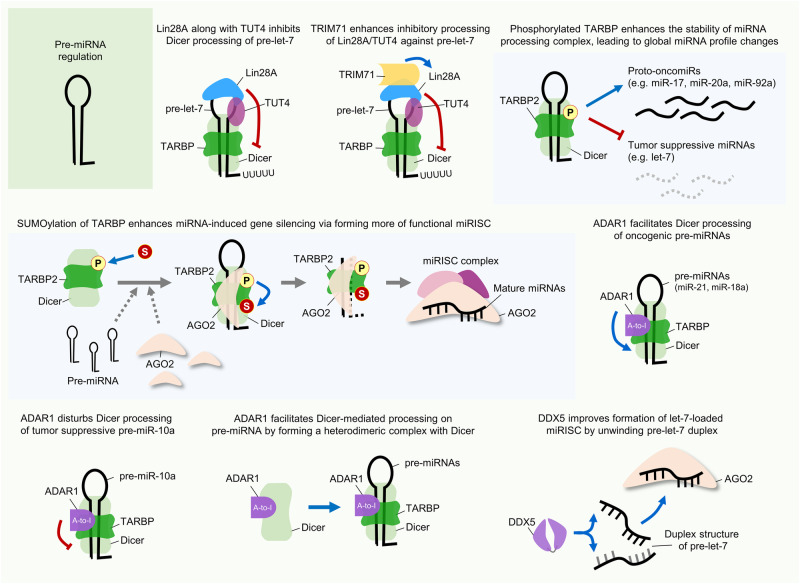
RBP action in pre-miRNA regulation. The figure illustrates several examples of the mechanism of action of RBP-mediated pre-miRNA regulation in cancer. RBPs have posttranscriptional mechanistic interactions with various pre-miRNAs and the Dicer-TARBP microprocessor complex. Consequently, RBP-triggered miRNA-dependent signaling pathways affect tumor progression. A detailed description of the mode of action is provided in the text.

Lin28 is closely associated with let-7 biogenesis, with effects on various forms ranging from pri- to pre-let-7, via potential shuttling between the nucleus and cytoplasm^49^. It can recognize G-rich sequences in the 3′-end stem‒loop of its target RNAs via its two domains^47,54,55^. Lin28 binds to pre-let-7 by recognizing the terminal loop and inhibits its further Dicer processing in the cytoplasm^56^. Then, Lin28 recruits the terminal uridyltransferase 4 (TUT4), which elongates the 3′-end of pre-let-7 with oligomeric (U), leading to its prompt degradation (Fig. 3)^57–59^. Lin28B can also bind to and exert an effect on pri-let-7 in a TUT4-independent manner (Fig. 2)^54,60^. Intriguingly, normally processed mature let-7 can also inhibit Lin28 expression by directly targeting its 3′UTR^51^. Thus, the relationship between Lin28 and let-7 can be considered a double-negative feedback loop in terms of regulating reciprocal functions.

Because let-7 has been reported to act as a tumor suppressor based on its targeting of several well-known oncogenes (K-Ras, c-Myc, and HMGA2)^61–63^, Lin28 is conversely considered to be an oncogenic factor and has been investigated in diverse human cancers. In breast cancer, Lin28 inhibits let-7a biogenesis, thereby facilitating EMT and promoting metastasis^64^. Additionally, dysregulation of Lin28 and let-7a expression is associated with resistance to radiotherapy in breast cancer^65^.

In lung cancers, Lin28 facilitates cell cycle progression and promotes cell proliferation by posttranscriptionally inhibiting let-7g biogenesis^66^. Another report also showed that Lin28 inhibition induces the upregulation of let-7a expression, resulting in repressed K-Ras expression and increased radiosensitivity in A549 lung cancer cells^67^. Lin28B but not Lin28A is predominantly overexpressed in HCC^68,69^. Overexpressed Lin28B promotes cell proliferation and EMT in vitro and facilitates tumorigenesis in vivo by suppressing the expression of let-7/miR-98^68,69^. Aberrantly increased Lin28B expression is also found in colon cancer and has been reported to be correlated with poor overall survival and a high recurrence rate, coinciding with reduced let-7a/b expression^70^.

In addition to the abovementioned studies on the regulatory effects of Lin28 and let-7 on cancers, other reports have also indicated that Lin28 can promote cancer progression by modulating let-7 biogenesis. Kong and colleagues showed that Lin28 can regulate the self-renewal capacity of prostate cancer cells by inhibiting let-7 production in combination with controlling the expression of stem cell markers (Sox2, Nanog, and Oct4)^71^. Another study in mice showed that Lin28 is affected by let-7; this relationship impedes normal kidney development^72^. That study also demonstrated that aberrantly high expression of Lin28 can eventually lead to the progression of Wilms tumor, the most common pediatric kidney cancer, by blocking normal postnatal differentiation of mouse embryonic kidney stem cells. Lin28-induced cancer progression was reportedly suppressed by let-7 supplementation, suggesting that Lin28 can promote Wilms tumor progression by at least partially inhibiting let-7 miRNA expression^72^.

Overall, the consistent evidence on Lin28 clarifies the importance of Lin28 modulating miRNAs such as let-7 during cancer progression and implies its utility in the development of therapeutic approaches.

## ELAVL1/HuR

ELAV-like RNA-binding protein 1 (ELAVL1), also known as HuR (human antigen R), is a representative RBP associated with the regulation of various RNAs. It is predominantly located in the nucleus; however, it can be translocated into the cytoplasm by shuttling in response to stressful cellular conditions^73^. Structural analysis revealed three RBDs (also called RRMs), RRM1 to RRM3, among which RRM1 and RRM2, near the N-terminus, are related to the recognition and binding of AU-rich elements (AREs)^74^. In general, ELAVL1/HuR is considered to be involved in RNA stabilization by binding to the ARE within the 3′UTR^73^. ELAVL1/HuR has been extensively studied in human diseases such as cancer after it was initially identified and cloned^75^. Emerging evidence has revealed the important functions of ELAVL1/HuR in regulating miRNAs, and these functions can be classified into two opposite categories: competitive and cooperative functions.

**Fig. 4:**
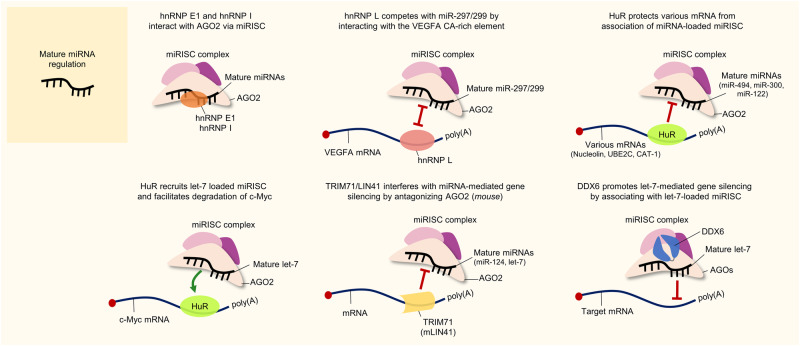
RBP action in mature miRNA regulation, miRISC formation, and miRNA targeting to the mRNAs. The figure illustrates several examples of the mechanisms of action of RBP-mediated mature miRNA regulation in cancer. RBPs have posttranscriptional mechanistic interactions with mature miRNAs, the key catalytic engine for miRISC formation (AGO2), and target mRNAs. Consequently, RBP-triggered miRNA-dependent signaling pathways affect tumor progression. A detailed description of the mode of action is provided in the text.

Regarding its competitive interactions with miRNAs, several studies have demonstrated that ELAVL1/HuR is involved in attenuating miRISC interactions with target RNAs (Fig. 4)^76,77^. For example, ELAVL1/HuR was reported to compete with miR-494 for the translation of nucleolin mRNA in cervical cancer cells^78^. It binds to the 3′UTR of nucleolin mRNA, thereby facilitating its translation by protecting it from interactions with the miR-494-loaded miRISC (Fig. 4). This competitive interaction between ELAVL1/HuR and miR-494 can partially affect cervical cancer cell proliferation by modulating nucleolin expression^78^. In addition, it was also found to disrupt the association of miR-300 with UBE2C mRNA, which is highly expressed in gastric cancer, eventually promoting cancer progression^79^. Notably, the binding sites of ELAVL1/HuR and miR-300 on the UBE2C 3′UTR overlap; thus, the authors proposed that ELAVL1/HuR can potentially protect UBE2C mRNA from being targeted by miR-300 (Fig. 4). Another study suggested that ELAVL1/HuR can relieve the effect of miR-122-induced CAT-1 mRNA degradation by binding to the CAT-1 3′UTR in HCC cells upon cellular stress^76^. Another related report revealed that CAT-1 mRNA is targeted and downregulated by miR-122; thereafter, its antiapoptotic and prosurvival effects can be attenuated in HCC cells^80^. Overall, these findings indicate that ELAVL1/HuR can compete with miR-122 for binding to CAT-1 mRNA, thus promoting cancer progression in HCC (Fig. 4)^6,76^.

Regarding its cooperative roles with miRNAs, ELAVL1/HuR can collaboratively function with let-7 to regulate c-Myc expression in cervical cancer cells^81^. ELAVL1/HuR interacts with the 3′UTR of c-Myc mRNA by recognizing its binding motif AREs, located near the let-7-binding site, eventually inhibiting c-Myc expression. Consequently, ELAVL1/HuR facilitates the recruitment and assembly of the let-7-loaded miRISC at the c-Myc 3′UTR, thus increasing the ability of let-7 to repress c-Myc expression (Fig. 4). Although the detailed mechanisms were not described in the study, the authors proposed that the association between ELAVL1/HuR and the c-Myc 3′UTR might lead to a conformational change in c-Myc mRNA that is susceptible to recognition by let-7^81^.

In addition, another study demonstrated the cooperative function of ELAVL1/HuR in breast cancer cells in a manner distinct from that identified in other similar studies. According to this study, miR-19b can negatively regulate P-glycoprotein (P-gp) expression and activity by repressing the translation of ABCB1 mRNA, which encodes P-gp^82^. The authors revealed that this regulation of miR-19b can occur through noncanonical interactions with the ABCB1 UTRs and that ELAVL1/HuR is indispensable for this noncanonical binding to the 3′UTR. Notably, the noncanonical binding site of miR-19b overlaps with the ELAVL1/HuR protein-binding sites within the ABCB1 3′UTR, and endogenous miR-19b was immunoprecipitated with endogenous ELAVL1/HuR. Therefore, it was suggested that miR-19b can be tethered to the 3′UTR of ABCB1 mRNA by binding to ELAVL1/HuR and suppress the translation of ABCB1^82^.

In addition to the aforementioned functions, ELAVL1/HuR can either directly or indirectly regulate miRNAs to affect the expression of their target mRNAs. ELAVL1/HuR was shown to promote gastric cancer cell proliferation in vitro and in vivo by negatively regulating miR-133b expression, in turn affecting the expression of the downstream gene CDC5L^83^. Since the detailed mechanisms underlying the negative modulation of miR-133b by ELAVL1/HuR were not clarified in the study, further investigations should be performed to identify the mode of action.

Overall, a comprehensive understanding of the omnidirectional mechanism of ELAVL1/HuR-miRNA interplay should be considered to improve the development of cancer therapeutics.

## KSRP

KSRP, also known as KHSRP (KH-type splicing regulatory protein), is a single-stranded RNA-binding protein located in both the nucleus and cytoplasm. KSRP contains four KH domains in the central region, which allow high-affinity binding to AREs (Fig. 1)^84^. KSRP was originally reported to control mRNA decay via direct binding to the ARE within the target mRNA 3′UTR^85^; however, subsequent studies demonstrated that KSRP can regulate mRNA stabilization by controlling miRNA biogenesis through binding to the G-rich terminal loops of pre-miRNAs and interacting with miRNA processing complexes containing Drosha and Dicer^33,86^. Therefore, KSRP-mediated mRNA and miRNA processing has been investigated in relation to various cellular events and was recently found to be involved in cancer progression. Notably, the roles of KSRP in miRNA processing in cancers have been found to differ by cancer type.

As described above in the hnRNP section, KSRP induces the biogenesis of the tumor-suppressive miRNA let-7a by competing with hnRNP A1 for binding to the terminal loop of pri-let-7a-1 (Fig. 2)^32,86^. Since the binding sites of these two RBPs, i.e., KSRP and hnRNP A1, overlap in the terminal loop of pri-let-7a-1^32^, the competition between KSRP and hnRNP A1 for pri-let-7a processing may contribute to the cellular expression of let-7a and to tumor progression in various cancers expressing let-7a. Moreover, KSRP was reported to attenuate invasive and metastatic features in non-small cell lung cancer (NSCLC) by negatively regulating oncogenic EGR3 mRNA stability through promoting the maturation of miR-23a, which targets EGR3 mRNA^87^. Similarly, KSRP can indirectly decrease metastatic potential by facilitating the expression of miR-192-5p, which can target several EMT-related factors in cancers^88^. Since EMT is a crucial factor in cancer progression and contributes to poor patient prognosis, the key evidence from this study could be utilized in cancer treatment for controlling metastatic traits.

On the other hand, it has also been reported that KSRP-mediated miRNA processing can promote cancer progression. For example, KSRP facilitates cancer growth in vitro and in vivo by indirectly inhibiting the mRNA expression of the tumor suppressor PTEN by promoting miR-26a maturation in small-cell lung cancer^89^. PTEN contains two binding sites for miR-26a in the 3′UTR, and the upregulation of miR-26a by KSRP results in increased targeting of PTEN mRNA. Since PTEN is reportedly associated with the PI3K/AKT signaling pathway in several cancers^90^, KSRP could be applied in PI3K/AKT signaling-targeted therapeutic strategies for small cell lung cancer. Similarly, KSRP can also accelerate the growth and increase the metastatic ability of esophageal squamous cell carcinoma cells by inducing the expression of three oncogenic miRNAs (miR-21, miR-130b, and miR-301a). Consequently, induction of these miRNAs results in repression of the translation of their target mRNAs PDCD4 (miR-21), TIMP3 (miR-21), and BMP6 (miR-130b and miR-301a), which are known to inhibit EMT in various cancers^91^. However, the detailed mechanism by which these miRNAs are modulated was not clearly identified in the study. The authors reported that none of the miRNAs contained conserved G-rich sequences within the terminal loop of their precursor form, even though the binding of KSRP to those miRNAs was confirmed in the experiments. Therefore, further investigations are needed to determine the exact mode of action and to evaluate whether KSRP and miRNA regulation by KSRP can be utilized as potential diagnostic or therapeutic targets in cancer.

In another study, KSRP was shown to augment the proliferation and metastatic features of clear cell renal cell carcinoma (ccRCC) cells by either directly or indirectly attenuating NEDD4L mRNA stability^92^. NEDD4L expression was reported to be significantly low in ccRCC and correlated with a positive prognosis. KSRP can abrogate NEDD4L mRNA expression by binding to the ARE within its 3′UTR and promoting the maturation of miR-629-5p, which binds to the 3′UTR of NEDD4L mRNA.

Taken together, these findings indicate that KSRP is closely related to diverse cancers in similar but distinct ways. Thus, approaches targeting KSRP for cancer treatment should be carefully applied to accurately control the intended oncogenic factors and minimize other unintended cellular events.

## TRIM71/LIN41

TRIM71 (tripartite motif-containing 71)/LIN41 (lineage variant 41), a member of the TRIM-NHL family, was initially discovered as a temporal cell fate regulator in *C. elegans* early development, and it is also a conserved target of the let-7 miRNA^93^. TRIM71 shares close structural similarities with other TRIM domain-containing protein family members in terms of the N-terminal tripartite motif (TRIM), which is composed of a RING domain, B-boxes, and coiled-coil regions (Fig. 1). Like other TRIM-NHL proteins, TRIM71 also has unique C-terminal features, including a filamin domain and an NHL (NCL-1, HT2A2, and LIN41) repeat motif (Fig. 1). All members of the TRIM-NHL family possess functional E3 ubiquitin ligase activity, which is critically dependent on the RING (Really Interesting New Gene) domain in the N-terminus^94,95^. The physiological functions of the other structurally defined motifs in TRIM71 besides the RING domain remain largely unknown.

As an example of indirect miRNA regulation by TRIM71, Rybak and colleagues showed that mLIN41 (a mouse homolog of TRIM71/LIN41) interacted with Dicer and AGOs. They reported that mLIN41 mediates the ubiquitination of AGO2 and impedes the silencing of the target mRNAs of let‑7 and miR‑124, potentially antagonizing the activity of AGO2. They also showed the cooperative action of mLIN41 with the pluripotency factor Lin28 in suppressing let‑7 activity (Fig. 4)^96^.

Another interesting observation was the indirect positive regulation of let-7 expression by TRIM71 through Lin28B polyubiquitination. Lee and colleagues demonstrated that TRIM71 negatively regulates Lin28B protein stability by catalyzing its polyubiquitination. Relative to its paralog Lin28A, Lin28B possesses a unique C-terminal sequence of ~50 amino acids that is critical for its TRIM71 interaction and subsequent polyubiquitination. Moreover, the N-terminal RING finger motif of TRIM71 is critical for protein‒protein interactions, the polyubiquitination of Lin28B, and consequent let-7 expression. Consistent with the stimulatory effect of TRIM71-mediated Lin28B polyubiquitination on let-7 expression, specific knockdown of TRIM71 led to downregulation of let-7 expression^94^. A recent report showed that TRIM71 also regulates let-7 expression and activity via two independent regulatory mechanisms. In this report, TRIM71 was stated to increase pre-let-7 degradation through its direct interaction with Lin28 and TUT4, thereby inhibiting let-7 maturation and indirectly promoting the stabilization of let-7 targets (Fig. 3). On the other hand, it was also reported that TRIM71 represses the activity of mature let-7 via its RNA-dependent interaction with AGO2^97^.

Regarding the direct regulation of RNA by TRIM71, Loedige and colleagues first demonstrated the conserved RNA-modulatory function of TRIM71/LIN41. The authors found that the NHL domain is necessary and sufficient for targeting TRIM71 to RNA, while the RING domain is not required for translational repression^98^. A recent report also showed that repression of the mRNA expression of the splicing regulator Muscleblind-like protein 1 (MBNL1) by TRIM71 is mediated by a 3′UTR hairpin. The authors identified a core set of TRIM71 targets, including MBNL1, in mice and humans and found that TRIM71-dependent repression of MBNL1 is required to maintain alternative splicing patterns in stem cells^99^. In line with the finding of direct RNA regulation by TRIM71, Liu and colleagues demonstrated that TRIM71 represses AGO2 mRNA translation in mouse embryonic stem cells. Blocking this repression led to a specific posttranscriptional increase of mature let-7 miRNA, resulting in let-7-dependent stemness defects and accelerated differentiation of stem cells^100^.

Despite such evidence of the RNA-modulatory role of TRIM71, there are very few reports regarding the regulation of miRNAs by TRIM71 during tumorigenesis. Yin and colleagues investigated the potential role of TRIM71 in tumorigenesis. Intriguingly, TRIM71 suppressed tumorigenesis in a manner dependent on its cellular ubiquitination target Lin28B. Moreover, subsequent modulation of let-7 and its posttranscriptional target HMGA2 was found to be essential for the antitumorigenic effect of TRIM71^95^. In regard to cancer-related aspects, TRIM71 was also reported to be a novel mutant p53-binding protein. TRIM71 overexpression is strongly associated with favorable prognosis, particularly in p53-mutated ovarian carcinomas^101^. Ren and colleagues demonstrated that TRIM71 expression in NSCLC is associated with a large tumor size, lymph node metastasis, a high TNM stage, and poor prognosis. The authors found that TRIM71 was more highly expressed in NSCLC cell lines than in their normal counterparts. TRIM71 was found to promote the proliferation of NSCLC cells through activation of the IκB-α/NF-κB signaling pathway. These results also suggested that TRIM71 plays a role in promoting the development of NSCLC^102^. The exact role of TRIM71 during tumorigenesis in conjunction with miRNA regulation is still under investigation.

## ARS2

Arsenic resistance protein 2 (ARS2), a nuclear protein encoded by the human SRRT gene (Serrate RNA effector molecule homolog)^103^, was initially identified as the gene that confers arsenite resistance on mammalian cells^104^. Functional studies have shown that ARS2 is highly expressed in proliferating cells, indicating that ARS2 plays a critical role in cell proliferation^105^. Primary amino acid sequence and crystal structure analyses revealed that human ARS2 has an N-terminal nuclear localization signal (NLS), a domain of unknown function (DUF3546), a central RNP/RRM motif for RNA substrate binding, a zinc finger (ZnF), and a C-terminal proline-rich unique nuclear cap-binding complex (CBC) binding motif (Fig. 1)^106,107^. It was reported that the C-terminus of ARS2 is also involved in RNA decay^107^. Structural analysis also confirmed that CBC directly interacts with ARS2, and the complex acts as a platform for the assembly of cotranscriptional complexes, that determine the fate of various RNA Pol II-generated transcripts^106^.

Plant and animal orthologs of ARS2 are well-known for their conserved roles in miRNA biogenesis^103^. ARS2 is also known to mediate the interaction between the CBC complex and the Drosha-DGCR8 heterodimeric pri-miRNA processing machinery during cell proliferation (Fig. 2)^105^. Biochemical studies indicate that ARS2 is a component of the nuclear CBC, which is required for miRNA biogenesis^105,108^. ARS2 depletion reduced pri-miRNA processing and the expression level of a number of miRNAs implicated in cellular transformation. Therefore, ARS2 is suggested to be a proliferation-inducible component of the CBC that also contributes to the regulation of RNA interference^105,108^. More interestingly, genetic deletion of ARS2 causes proliferation arrest and bone marrow hypoplasia^105^.

In addition to its role in miRNA processing, ARS2 maintains neural stem cell identity and self-renewal capacity via direct transcriptional activation of Sox2, as reported by Andreu-Agullo and colleagues^109^. Regarding the transcriptional regulatory role of ARS2, Yin and colleagues showed that ARS2 directly activates its transcriptional target monoacylglycerol lipase to regulate the self-renewal and tumorigenicity of glioma stem cells (GSCs)^110^. Another report also showed that knockdown of ARS2 suppressed tumor growth in an orthotopic glioblastoma xenograft model and significantly prolonged the survival of tumor-bearing mice. In addition, ARS2 knockdown reduced the expression of miR-6798-3p, which caused upregulation of p53 and p21^111^. Overall, ARS2 may facilitate cancer progression by inhibiting p53/p21-mediated apoptosis via the upregulation of miR-6798-3p.

Despite its miRNA-modulatory role, ARS2 is frequently overexpressed in various cancers. ARS2 is overexpressed in patients with HCC and is associated with poor overall survival, suggesting that it is a potential prognostic marker for HCC development^112^. In another study, ARS2 was found to be overexpressed in human cholangiocarcinoma (CCA) specimens compared with paired normal tissues. The diagnostic sensitivity and specificity of ARS2 mRNA levels in human CCA were reported to be very high, which highlights the clinical importance of ARS2 as a diagnostic and prognostic marker in CCA^113^. ARS2 was also found to be overexpressed in AML cell lines and bone marrow samples from patients with AML and to be correlated with poor overall survival, further highlighting its clinical importance^114^. These studies suggest that the miRNA-modulatory role of ARS2 may be important and that targeting strategy of ARS2 may be a therapeutic option for various diseases, especially cancers.

## TARBP

TARBP (transactivation response RNA-binding protein, TRBP) contains two double-stranded RNA-binding domains (dsRBDs) for RNA-binding affinity and one C-terminal Medipal region containing a C4 domain for protein‒protein interactions (Fig. 1)^115^. TARBP has two homologs, TARBP1 and TARBP2, which are structurally identical except for the presence of 21 amino acids at the end of the N-terminus^115^. In regard to the multifarious roles of TARBP, it was originally identified as a transactivator that binds to the TAR region of the HIV-1 RNA genome and affects viral synthesis^116^; thereafter, TARBP was shown to function with Dicer as an integral component of the RISC in RNA interference, especially miRNA interference^115,117,118^. Furthermore, it has been found to be involved in modulating cancer-related genetic and proteomic statuses, such as aberrantly dysregulated expression and translational modification^115^, and to exert cancer-related physiological effects, including morphological alterations, promotion of cell proliferation, and dysregulation of cellular miRNA profiles^119,120^. However, it has conflicting roles in various cancers, and these roles have not yet been fully established. This section focuses on diverse lines of evidence indicating that TARBP could function as a notable factor in cancer progression by mediating miRNA processing.

Regarding the effects of its modification, TARBP is phosphorylated via the MAPK/Erk pathway, which increases the stabilization and expression of miRNA processing complex composed of Dicer-TARBP, and posttranscriptionally regulates cancer-related miRNA production (Fig. 3)^120^. In terms of alterations in miRNA production, there is one interesting anomaly. Whereas the levels of proto-oncogenic miRNAs, such as miR-17^121^, miR-20a^122^, and miR-92a^123^, were increased by phosphorylated TARBP, the level of only the tumor-suppressive let-7 miRNA was decreased under the same conditions. Moreover, these alterations led to increased proliferation and viability of cervical cancer cells. Through pharmacological inhibition of the MAPK/Erk pathway to inhibit TARBP phosphorylation, the authors additionally confirmed phosphorylated TARBP-mediated miRNA profile changes in human cervical, gastric, and lung cancer cells.

Another study reported the effect of TARBP2 SUMOylation. TARBP2 is SUMOylated largely by the SUMO1 protein, and this modification can be augmented by TARBP2 phosphorylation^124^. TARBP2 SUMOylation can increase the gene targeting efficiency of miRNAs, not miRNA production, by enhancing its binding affinity for pre-miRNAs, stabilizing AGO2, and finally forming more of functional RISC. Consequently, TARBP2 SUMOylation likely facilitates miRNA-induced gene silencing by recruiting more AGO2 to form a RISC-loading complex (Fig. 3). Moreover, TARBP2 SUMOylation was found to be involved in the progression of lung cancer cells both in vitro and in vivo.

Regarding the effects of its aberrant expression, TARBP1 is highly expressed in several cancers, including HCC and NSCLC^125,126^. High expression of TARBP1 has consistently been found to be significantly associated with high histological grade, high clinical stage, and poor patient overall survival. However, the precise mechanism by which TARBP1 affects cancer progression was not fully investigated in the related studies. One study revealed that highly expressed TARBP1 can promote the translation of glutamine transporters by inducing Gm18 modification of tRNA, thereby increasing the efficiency of glutamine uptake by proliferating liver cancer cells^126^. Although no specific miRNA-mediated roles for TARBP1 have been identified, it could be presumed that the RNA methyltransferase activity of TARBP1 might affect Dicer cleavage activity by modulating the 5′-ends of pre-miRNAs, as previously reported for BCDIN3D^127^.

In contrast, TARBP2 has been shown to be differentially expressed in various cancers. It was reported to be downregulated in HCC, colorectal cancer, gastric cancer, and Ewing sarcoma^128–130^ but upregulated in prostate cancer, breast cancer, lung cancer, cutaneous malignant melanoma, and adrenocortical carcinoma^131,132^. Several studies have speculated that TARBP2 functions via miRNA-independent and mRNA-modulating mechanisms. Li and colleagues suggested a TARBP2-mediated miRNA-regulatory mechanism in HCC^128^. They showed that TARBP2 can facilitate miR-145 maturation, leading to repressed expression of the miR-145 target SERPINE1 and affecting cancer progression mediated by the HIF-1 signaling pathway.

In addition to the abovementioned findings, several notable studies have suggested that TARBP2-mediated miRNA regulation can restore global miRNA profiles in cancer cells and consequently affect cancer progression-related features^129^. De Vito and colleagues reported that TARBP2 depletion in Ewing sarcoma family tumor (ESFT) cells can promote self-renewal capacity and tumorigenesis by changing the miRNA profiles of these cells to be similar to those of ESFT cancer stem cells. This report suggested that TARBP2 can inhibit the stemness features of ESFT cells by maintaining their miRNA profiles. Furthermore, this mechanism was validated using enoxacin, a small-molecule antibacterial fluoroquinolone that promotes TARBP2 activity through an interaction^129,130^. Melo and colleagues reported that the binding of enoxacin to TARBP2 results in global miRNA profile changes, leading to notable increases in the levels of tumor-suppressive miRNAs in various cancer cells^130^. Since the abundance of pre-miRNAs was decreased and that of mature miRNAs was increased, this effect could possibly be attributed to the role of TARBP2 in miRNA processing activated by enoxacin treatment. Based on this report, it was confirmed that the expression of TARBP2-dependent miRNAs (miR-143 and miR-145) was inversely correlated with stemness features; thereby the potential as a therapeutic target was validated in cancers.

Overall, the aforementioned evidence indicates the significance of TARBP in therapeutically controlling miRNA expression and activity in terms of cancer treatment and implies that TARBP activity might be efficiently controlled with new or preestablished drugs targeting its modification and activity. However, prior to evaluating the reproducibility of these results, it should be carefully considered that most miRNAs have dual functions and play opposing roles in various cancers.

## ADAR

ADAR (adenosine deaminase acting on RNA) contains dsRBDs and a catalytic deaminase domain, and the human genome encodes three members of the ADAR family, which are the catalytically active ADAR1 and ADAR2 and the predominantly inactive ADAR3 (Fig. 1)^133,134^. ADAR1 and ADAR2 are ubiquitously expressed in various tissues, and ADAR2 but not ADAR1 is highly expressed in the brain. ADAR3 is known to be expressed exclusively in the brain^134^. ADAR promotes the generation of inosine (I) via deamination of adenosine (A), a process called A-to-I RNA editing, by binding to double-stranded RNA^133^. A-to-I RNA editing commonly occurs in diverse cancers and plays important roles in the uncontrolled growth of cancer cells via various mechanisms^133,135^. Since pri- and pre-miRNAs have stem‒loop structures, which could be considered as ‘double-stranded RNA’, ADARs can change their structure, and this change is followed by altered Drosha/Dicer cleavage and RISC loading. Consequently, it affects miRNA biogenesis and even alters target mRNA recognition^136,137^. As expected, accumulating studies have revealed the miRNA editing effects of ADARs, especially ADAR1 and ADAR2, in various cancers^138–140^.

ADAR1-mediated miRNA regulation has been reported in a wide range of cancer types. For example, ADAR1 plays oncogenic roles in gastric cancer through suppressing interferon signaling mediators (IRF9 and STAT1) by promoting the biogenesis of the miR-302/367 cluster, especially in Drosha processing step (Fig. 2)^141^. Since miR-302a-3p can target IRF9 by directly binding to its 3′UTR and miR-302-5p can weakly bind to STAT1 and indirectly regulate its expression, ADAR1-mediated miR-302a regulation might influence gastric cancer in an integrative manner. Furthermore, ADAR1 plays oncogenic roles in oral squamous cell carcinoma (OSCC). It can directly interact with Dicer in the cytoplasm and augment the biogenesis of several oncogenic miRNAs in OSCC, including miR-21, miR-18a, miR-210, miR-155, miR-181a and miR-19a (Fig. 3)^142^. Although the specific downstream targets of these miRNAs were not identified in the related study, they could be expected to inhibit tumor suppressor genes in OSCC. It was also confirmed that ADAR expression is correlated with poor patient overall survival, and it promotes EMT and stemness features in vitro and tumorigenesis in vivo.

Similarly, ADAR1 was also reported to promote cancer progression in chordoma, which is a rare malignant bone tumor^143^. However, it promotes tumorigenic progression in an opposite manner to those noted in the two previous reports in which oncogenic miRNA maturation was shown to be facilitated by ADAR1. Overexpressed ADAR1 in chordoma disturbs the biogenesis of tumor-suppressive miRNAs (miR-10a and miR-125a) during the processing steps by Dicer and Drosha, respectively (Fig. 2 and Fig. 3)^143^. Thus, increases in the levels of pre-miR-10a and pri-miR-125a were inferred under the same conditions. Consequently, the reduced expression of miR-10a and miR-125a upregulated the expression of their oncogenic target genes, namely, HOXA1 and ERBB2. Similarly, ADAR1 can impair the biogenesis of the tumor-suppressive miRNA let-7 in leukemia by attenuating its Drosha cleavage (Fig. 2), thereby increasing self-renewal capacity and the gene expressions related to this process in leukemia stem cells^140^. In addition, ADAR1 was reported to increase cell proliferation and self-renewal capacity in vitro and tumorigenesis in vivo in lung cancer^144^. Aberrant gene amplification results in high expression of ADAR1 in NSCLC cells, thus contributing to the increased RNA editing frequency of DNA repair-associated NEIL1 mRNA and the cancer stemness-associated miR-381^145^. In contrast, significantly low ADAR1 expression was found in highly metastatic melanoma cell lines. ADAR1 can bind and edit pri-miR-455, further attenuating the binding capacity of Drosha and suppressing miR-455 maturation (Fig. 2). Moreover, the inhibitory effect of mature miR-455-5p on CPEB1 expression is impaired by ADAR1 editing, and edited miR-455 loses its original oncogenic effect and instead suppresses tumor progression and lung metastasis in melanoma^146^.

ADAR2-mediated miRNA regulation in cancer has been intensively investigated not only in brain tumors but also in other cancers. For example, functionally impaired ADAR2 can aggravate glioblastoma progression by affecting cellular miRNA profiles^147^. When functioning normally, ADAR2 represses oncogenic miR-21 and miR-221/222 in the brain by interfering with Drosha processing of their pri-miRNAs through the editing of stem‒loop structures (Fig. 2). In glioblastoma, its normal catalytic editing activity is defective; therefore, disrupted function of ADAR2 promotes glioblastoma progression. The functionally defective effects of ADAR2 in glioblastoma was also confirmed in other studies. Impairment of ADAR2 contributes to the accumulation of abnormally unedited miR-376a-3p through reduced modification of pri-miR-376a1 (Fig. 2)^148^. Unedited miR-376-3p cannot bind to its original target, AMFR, but associates with the alternative target RAP2A. Eventually, glioblastoma cells are reprogrammed into more progressive and invasive in patients. In addition, ADAR2 can abrogate the binding between pri-miR-214 and its antisense RNA transcripts by modifying the nucleotides of the transcripts in HCC^139^. The increased number of mismatches prevents the normal processing of pri-miR-214 into mature miR-214. In turn, the reduced level of mature miR-214 can lead to increased expression of its target gene Rab15, which is a member of the Ras oncogene family. Since ADAR2-overexpressing hepatocytes exhibit miRNA alterations similar to those observed in HCC cells, ADAR2 could be expected to act as a promoter in HCC progression.

Along with these editing-dependent effects of ADARs, other regulatory functions of ADARs, including editing-independent effects, have been suggested. For example, ADAR1 can also form a heterodimeric complex with DGCR8 and possibly impede Drosha-DGCR8 complex formation, hence altering miRNA expression profiles in melanoma. ADAR1 expression is reportedly reduced following metastatic progression in melanoma, and the reduction in ADAR1 expression alters miRNA expression profiles to facilitate cell proliferation in vitro and tumorigenesis in vivo^149^. Furthermore, ADAR1 can interact with Dicer to form another heterodimeric complex, further facilitating Dicer-mediated cleavage of pre-miRNAs and increasing RNA silencing via the miRISC (Fig. 3)^150^. The upregulated miRNAs can affect the expression of their target genes. Although this finding has not been verified in cancer, aberrantly regulated miRNAs may influence tumorigenesis. In addition, ADAR2 was found to disrupt Drosha-mediated cleavage of pri-miR-376a2 in a catalytic activity-independent manner^151^.

Collectively, these diverse functions of ADAR in miRNA regulation imply its high potential for application as a multifariously effective target in cancer treatment. Since the roles of ADARs vary depending on the type of cancer, the mechanisms of action of each ADAR should be clearly demonstrated and identified in each relevant cancer prior to the development of therapeutic strategies for ADARs.

## DEAD-box RNA helicases

The DEAD-box RNA helicase family is the largest family of RNA helicases known to generally unwind RNA duplex structures by dissociating hydrogen bonds between nucleic acids^152,153^. Members of this family are highly conserved RBPs that contain common core structures with multiple conserved motifs, including D-E-A-D (Asp-Glu-Ala-Asp) signature sequences, which are associated with their ATP-binding and RNA unwinding functions (Fig. 1)^152,153^. The other functions are attributed to other structural features, such as the terminal domains containing RNA/protein-binding sites^154^. According to accumulating reports, DEAD-box RNA helicases have been consistently demonstrated to play key roles in diverse aspects of RNA metabolism, such as RNA processing, turnover, transcription, and translation by rearranging complex RNA structures^154,155^. Since cancer cells largely demand aberrantly upregulated translation of oncogenic mRNAs to promote their uncontrolled proliferation^155^, targeting DEAD-box RNA helicases could be considered an encouraging strategy in cancer therapeutics^156^. Moreover, in addition to playing these ATPase activity-dependent roles, DEAD-box RNA helicases can multifariously influence cancer cell fates by regulating other factors, such as miRNAs, during cancer progression^154,156,157^. In this respect, this section focuses on several representative DEAD-box RNA helicases associated with miRNA regulation in cancer.

DDX3, also known as DDX3X or DBX, has been studied in various human diseases and pathological processes, including viral infection, inflammation, and cancer^158^. In recent decades, its key roles in cancer development and progression have continually been revealed. In particular, there have been some reports of its direct and indirect miRNA-regulatory roles in cancers. For example, DDX3 can change the expression profiles of a subset of miRNAs by binding to the Drosha-DGCR8 microprocessor complex and increasing its processing activity, further facilitating pri-miRNA processing and miRNA maturation (Fig. 2)^159^. In the related study, the authors demonstrated the role of DDX3 in promoting the biogenesis of a subset of miRNAs (miR-1, miR-141, miR-145, miR-19b, miR-20a, and miR-34a). Correlation analysis of the expression of these miRNAs and DDX3 in various cancers in The Cancer Genome Atlas (TCGA) database revealed that the related miRNA alterations might be caused by alterations in the expression of DDX3 in most cancers. Similarly, another report demonstrated that DDX3 binds to pri-miR-20a and increases its stability, thus facilitating miR-20a biogenesis in K562 leukemia cells (Fig. 2)^160^. Since the detailed effects on cancer-related phenotypes were not described in these two reports, further investigation is required to verify the precise role of DDX3 in each cancer type. Furthermore, DDX3 reported to attenuate cancer stem cell-like characteristics in HCC by interfering with DNMT3A binding and the induction of DNMT3A-mediated methylation on the promoter of tumor-suppressive miRNAs, thus facilitating the transcription of these miRNAs (i.e., miR-200b, miR-200c, miR-122, and miR-145)^161^. Along with these results, DDX3 expression was found to be very low in high-grade HCC, and low DDX3 expression is associated with poor patient prognosis. Therefore, strategies that maintain high expression of DDX3 could be utilized in HCC therapeutics. Whereas the related report suggested the methylation-blocking mechanism of DDX3, another report addressed the demethylation-inducing mechanism of DDX3, and this evidence could mutually support the establishment of epigenetic RNA-modulating roles. DDX3 was also shown to directly bind to ALKBH5, which is an N6-methyladenosine (m^6^A) RNA demethylase, and to have a concomitant physical association with AGO2^162^. These multiple interactions of DDX3 could lead to increased demethylation of miRNAs by ALKBH5. As such, DDX3 can act as either a tumor suppressor or an oncogenic factor. This is possible through its unusual functions of forming protein complexes with other protein partners, and these interactions could alter cancer-related molecular signaling pathways or the expression of miRNAs that mediate cancer progression or suppression^154,163^.

DDX5, also known as p68, is regarded as a prototypic member of the DEAD-box RNA helicase family since it has amino acid sequences largely homologous to those of the initially identified RNA helicase eIF4A^164^. DDX5 was found to be involved in several biological functions as transcriptional coactivator and to be associated with cancer progression, and it has also been demonstrated to have important roles in RNA metabolism, including miRNA processing and transcription^154^. In this regard, various studies have indicated the miRNA regulation-mediated roles of DDX5 in cancer. It has been consistently observed that DDX5 can function in the nuclear maturation of miRNAs by interacting with the Drosha-DGCR8 complex or other RNA-associated functional proteins. For example, DDX5 is involved in LMTK3 (Lemur tyrosine kinase 3)-dependent miRNA processing in breast cancer^165^. DDX5 enables LMTK3 to facilitate the processing of pri-miRNAs (pri-miR-34a, pri-miR-182, and pri-miR-196-a2) for maturation, and upregulated miR-34a and miR-182 can target and inhibit LMTK3 expression, consequently suppressing the oncogenic effects of LMTK3 in breast cancer. In contrast, it was reported that DDX5 is involved in TGF-β (transforming growth factor-β)- and BMP (bone morphogenic protein)-specific SMAD signaling-induced oncogenic miR-21 maturation by forming a protein complex with Drosha-DGCR8 in breast cancer cells (Fig. 2)^166,167^. Moreover, DDX5 can be phosphorylated by PAK5 [p21 (RAC1)-activated kinase 5], and its stability can then be enhanced through phosphorylation-induced SUMOylation^168^. These PAK5-mediated modifications consequently increase oncogenic miR-10b biogenesis by augmenting the interaction of DDX5 with the Drosha-DGCR8 complex, facilitating pri-miR-10b cleavage and maturation in breast cancer (Fig. 2). Accordingly, increased miR-10b expression may promote breast cancer progression and metastasis. DDX5 can also directly modulate miRNA biogenesis through its helicase activity. It unwinds the pre-let-7 duplex structure and facilitates the incorporation of the let-7 guide strand into the RISC, indicating that it is required for oncogene silencing mediated by the tumor-suppressive let-7 in cancers (Fig. 3)^169^. Overall, DDX5 has been found to be involved in cancer progression through either tumor-suppressive or oncogenic mechanisms, depending on its interaction partners, even in the same cancer type.

DDX6, also termed Rck/p54, was first identified at a chromosomal breakpoint in lymphoma cells and was subsequently reported to be highly expressed in most cancer cell lines^170–173^. For this reason, it has been investigated as a proto-oncogene in various cancers; however, its detailed mechanism of action has not been fully established^154^. In particular, the miRNA-regulatory mechanisms of DDX6 are relatively deficient compared to those of other DEAD-box RNA helicases. Among the numerous cancer-associated studies, reports revealed that DDX6 can affect cancer progression by controlling miRNA biogenesis or activity. Iio and colleagues showed that DDX6 posttranscriptionally inhibits miR-143/145 expression by decreasing the stability of their host gene, NCR143/145 (MIR143HG), which encodes both miRNAs^174^. DDX6 is mainly localized in processing bodies (P-bodies) in gastric cancer cells, and a reduction in the DDX6 abundance in P-bodies leads to an increase in miR-143/145 expression by stabilizing the host pri-miRNA transcript NCR143/145. Considering that the expression of miR-143/145 is low in most cancer types and that NCR143/145 is also difficult to detect experimentally, increasing the expression of these miRNAs along with that of their host genes could be a promising strategy for gastric cancer treatment. In addition, Chu and colleagues reported that DDX6 is required for the repression of translation mediated by the tumor-suppressive miRNA let-7 in cervical cancer cells by directly interacting with AGO1 and AGO2, thereby forming the let-7-mediated miRISC (Fig. 4)^175^. Based on this evidence, DDX6 may be associated with miRNA-regulatory roles in cancer progression. Although it is clearly deficient to delineate comprehensive mode of action, it would be more thoroughly investigated in various cancers henceforward.

DDX17, also known as p72, is a paralog of DDX5 and has functions that are partially redundant with those of DDX5^176^. Therefore, it has also been investigated in the context of miRNA metabolism as a cofactor of the Drosha-DGCR8 microprocessor complex^154,177^. Regarding the mechanism by which DDX17 modulates miRNAs, it is generally considered to recognize and bind to the stem‒loop structure of pri-miRNAs, facilitating their further processing^178^. For example, DDX17 is involved in the maturation of neuronal miRNAs by acting as a direct cofactor of REST (repressor element 1-silencing transcription factor), which targets neuronal genes, during neuronal differentiation^179^. DDX17 regulates the nuclear maturation of pri-miR-26a2 during the early stages of neuronal differentiation, and interestingly, processed mature miR-26a can reciprocally target DDX17 in neuroblastoma cells (Fig. 2). Moreover, it interacts with REST at the promoters of REST target genes, enhancing neuronal gene regulation. Since the neuronal differentiation status is a critical factor in neuroblastoma development, DDX17 can be targeted for the regulation of differentiation in neuronal cancer therapy. In addition, DDX17 influences the biogenesis of various miRNAs along with the YAP protein, the downstream target of the tumor-suppressive Hippo signaling pathway in cancer^180^. At a low cell density, DDX17 is dissociated from the Drosha-DGCR8 microprocessor complex and sequestered by activated YAP; thereafter, miRNA biogenesis is globally abrogated via inhibition of pri-miRNA processing. Since the miRNAs repressed by YAP include oncogenic Myc-targeting miRNAs such as let-7 and miR-34a, posttranscriptionally induced Myc protein expression might drive accelerated cancer progression and tumorigenesis (Fig. 2). Moreover, DDX17 was reported to augment cancer-associated features by inhibiting miRNAs. DDX17 can promote EMT and metastasis in colorectal cancer (CRC) by mediating the miR-149-3p/CYBRD1 axis^181^. The expression of the tumor-suppressive miR-149-3p is reduced by DDX17, resulting in increased expression of its target CYBRD1. However, the exact mechanism by which DDX17 inhibits miR-149-3p was not elucidated in the related study; thus, further investigations are needed to determine the repressive effects of DDX17 on miRNAs in CRC.

Furthermore, DDX17 has been shown to modulate miRNAs through additional mechanisms^177^. DDX17 also controls the miRNA abundance by stabilizing AGO2 and posttranscriptionally regulating its expression in a proteasome-dependent manner in cervical cancer cells^182^. Moreover, the RMFQ (R250, M254, F256, and Q259) groove in the DDX17 catalytic core can recognize specific sequence motifs in pri-miRNAs, remodel its 3′ flanking structure, and facilitate pri-miRNA processing (Fig. 2)^183^. Additionally, the N-terminal extended domain of DDX17 can interact intramolecularly with the DEAD-box domain, attenuating its ATPase activity. These findings can provide a comprehensive understanding of the molecular mechanisms of the miRNA regulation by DDX17 and further support the exploration of specific miRNA-regulatory therapeutic strategies for cancer.

DDX23, also known as PRP28, is a U5 snRNP-specific 100-kDa protein first identified in the spliceosome complex^184^. Its amino acid sequences are highly conserved from *C. elegans* to humans^185^. Unlike the above-described DEAD-box RNA helicases, DDX23 was not actively investigated for its miRNA-regulatory roles. However, there are a few related reports regarding its possible functions. For example, the results of genome-wide RNAi screening suggested that DDX23 may be involved in let-7 biogenesis^186^, and another report demonstrated the role of DDX23 in pri-let-7 processing in *C. elegans*^187^.

Furthermore, the pri-miRNA processing function of DDX23 has been investigated in human cancers. DDX23 can promote cell proliferation and invasive features in glioblastoma by promoting oncogenic miR-21 maturation^188^. DDX23 posttranscriptionally facilitates pri-miR-21 processing by interacting with the Drosha-DGCR8 microprocessor complex (Fig. 2). The role of DDX23 in pri-miR-21 processing is primarily mediated by its intrinsic helicase activity, and the inhibition of its helicase activity for glioblastoma treatment was experimentally demonstrated by using ivermectin, a viral helicase inhibitor. The effect of the drug was investigated and found to be mediated by DDX23 and linked with the processing of pri-miR-21. These results provide insight into the advantages and potential of pharmacologically targeting the miRNA-modulating helicase activity of DDX23 in cancer therapeutics.

## Dysregulation of exoribonucleases modulating miRNA in cancer

Ribonucleases are able to degrade specific RNAs at the transcriptional and posttranscriptional levels. Their functions were previously considered to be simply destroying ribonucleotides and inducing cytotoxic effects by degrading RNA molecules; however, they have been discovered to play critical roles in processes such as splicing and RNA processing even in cancers^189^. They can be characterized into two large families based on the location at which they initiate the cleavage of target RNAs: endoribonucleases and exoribonucleases. Since each of these two families contains a number of members, this review focuses on the various exoribonucleases to more comprehensively delineate primary research findings according to the purpose of this review. In addition, exoribonucleases can be classified as 5′ to 3′ (5′–3′) or 3′ to 5′ (3′–5′) exoribonucleases based on their directionality of cleavage and/or degradation of target RNAs. Since several notable exoribonucleases can broadly impact gene expression through miRNA processing (Table 2), it is evident that they or their aberrant function may have implications for cancer progression. This section reviews several representative exoribonucleases that reportedly affect cancer progression by directly trimming/degrading miRNAs or indirectly regulating miRNAs (Table 2).

**Table 2:** Summary of representative exoribonucleases modulating miRNAs associated with various cancers and other noteworthy biological events or species.

Directionality	Exoribonuclease	Target miRNAs associated with cancer	Mechanisms of miRNA modulation	Functions associated with miRNA modulation	Ref.
5′–3′	XRN1	Tumor-suppressive miRNAs (miR-363, miR-101, miR-128); prostate cancer	Pre-miRNA degradation	Involved in the turnover of several tumour-suppressive miRNAs at the precursor level by forming a processing complex with IFIT5, which binds to the pre-miRNAs by recognizing the 5′-end overhang structure	^15^
		miR-34a; prostate cancer	^a^N.D.	Maintains the aberrantly high expression level of XRN1 via a positive feedback loop, in which XRN1 degrades miR-34a, which targets the androgen receptor, and the subsequent androgen receptor upregulation attenuates miR-204 targeting of XRN1	^195,196^
		ex-miR-223-3p; lung cancer	N.D.	Promotes the expeditious decay of ex-miR-223-3p, which inhibits the expression of its target FOXO1	^197^
		miR-382 (HEK293T); HIV-1 provirus latency	Mature miRNA degradation	Selectively degrades miR-382 and affects its stabilization	^192^
		miR-241, miR-58, miR-73 (*C. elegans*)	Passenger strand degradation	Degrades passenger strands of certain miRNAs (miR-241, miR-58, and miR-73) and affects miRNA turnover	^198^
		miR-277-3p (*Drosophila*)	miRNA maturation (not confirmed)	Affects the maturation of miR-277-3p at the posttranscriptional level	^199^
	XRN2	miR-10a; lung cancer	Pre-miRNA processing	Directly binds to pre-miR-10a and promotes its maturation in a Dicer-independent manner	^16^
		Tumor-suppressive miRNAs (let-7 family); lung cancer, HCC, and glioblastoma	Mature miRNA degradation	Degrades several tumor-suppressive miRNAs (let-7 family) after dissociation from AGO2/miRISC via a putative unidentified miRNA-releasing factor	^204^
		let-7, miR-60 (*C. elegans*)	Mature miRNA degradation	Affects the accumulation of mature miRNAs by facilitating miRNA dissociation from the AGO protein	^205^
		Tumor-suppressive miRNAs (let-7c, let-7g); neuroblastoma	Mature miRNA degradation	Actively degrades a subset of tumor-suppressive miRNAs, further contributing to cancer progression	^206^
3′–5′	PARN	miR-21	Mature miRNA trimming/degradation	Degrades 3′-end oligo(A) tails of miR-21 that were adenylated by PAPD5	^212^
		miR-451; chronic myeloid leukemia	Pre-miRNA trimming	Trims the 3′-end of the AGO2-cleaved pre-miR-451 intermediate into the mature length	^213^
		miR-362; cervical cancer	Mature miRNA trimming	Efficiently decreases the length of miRNAs by trimming the 3′-extensions of miRNAs that are derived from the genome or attached by terminal nucleotidyltransferases	^210^
		miR-122; HCC	Mature miRNA degradation	Destabilizes 3′-adenylated miR-122 via deadenylation	^214^
		Several miRNAs targeting p53 (miR-380-5p, miR-1285, miR-92, miR-214, miR-331, miR-25, miR-3126); cervical cancer	Pre-miRNA and mature miRNA trimming	Stabilizes miRNAs by removing oligo(A) tails of mature- or pre-miRNAs that were added by the poly(A) polymerase PAPD5	^215^
		miR-7; glioblastoma	Mature miRNA degradation	Degrades EGFR-targeting miR-7 containing a U-rich nucleotide composition at the 3′-end, therefore positively modulating EGFR expression in GSCs	^19^
	ERI1	miR-20a, let-7a, miR-16, miR-19b	Mature miRNA degradation/trimming	Trims AGO-associated full-length miRNAs to 14-nt or shorter tiny RNAs	^222^
	DIS3	miR-252-5p (*Drosophila)*	Mature miRNA degradation	Specifically degrades the mature miRNA, not the precursor form, in the cytoplasm during development	^228^
		miR-982-5p (*Drosophila)*	Pre-miRNA processing	Involved in the normal processing of pre-miRNAs into the mature miRNA form	^228^
		miR-31, miR-106b (mouse embryonic fibroblasts)	Pre-miRNA degradation	Degrades pre-miRNAs in cooperation with TUT4/7 based on its distinct preferences for pre-miRNAs, such as those with > 2 nt in the 3′-overhang	^229^
		let-7; multiple myeloma	Mature miRNA protection (indirect)	Promotes the maturation of let-7 miRNA by degrading Lin28B mRNA, which inhibits Drosha-mediated processing in a TUT-independent manner	^230^
	DIS3L/DIS3L2	Several miRNAs targeting p53 (miR-380-5p, miR-1285, miR-92, miR-214, miR-331, miR-25, miR-3126); cervical cancer	Pre-miRNA and mature miRNA degradation	Degrades pre-miRNAs uridylated by TUTs (DIS3L2) or adenylated by PAPD5 (DIS3L) and mature miRNAs adenylated by PAPD5 (DIS3L/DIS3L2)	^215^
	DIS3L2	let-7 (mouse embryonic stem cells)	Pre-miRNA degradation	Promotes the degradation of oligo-uridylated pre-let-7 mediated by TUTs	^231,232^

^a^N.D.; not described.

## 5′–3′ exoribonuclease XRN1

**Fig. 5:**
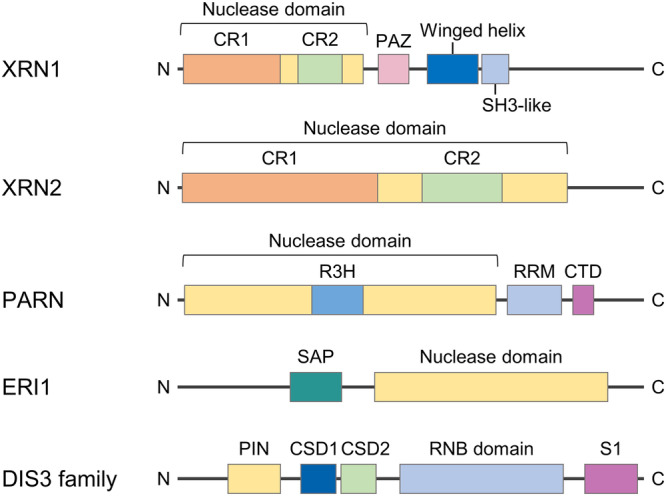
Schematic diagram of the structure of miRNA-modulating exoribonucleases with functional domains and motifs. The 5′–3′ exoribonuclease XRN1 contains two conserved regions, CR1 and CR2, within the nuclease domain, a PAZ domain, a winged helix domain, and an SH3-like motif. The 5′–3′ exoribonuclease XRN2 also contains CR1 and CR2 within its nuclease domain. The 3′–5′ exoribonuclease PARN contains an R3H (arginine-three amino acid residues-histidine) motif within the nuclease domain, an RRM, and a CTD (C-terminal domain). The 3′–5′ exoribonuclease ERI1 contains a SAP domain for nucleic acid binding and a nuclease domain. In the DIS3 3′–5′ exoribonuclease family, DIS3 and DIS3L contain a PilT N-terminal (PIN) domain, but DIS3L2 does not; all DIS3 family members contain two tandem cold-shock domains (CSD1 and CSD2) for nucleic acid binding, a ribonuclease II (RNB) catalytic domain that supports RNA degradation, and an S1 domain in the C-terminal region that is known to confer substrate RNA binding. A detailed description of various exoribonucleases, with their functional domains and motifs, is provided in the text.

XRN1 is one of the most extensively investigated 5′–3′ exoribonucleases and has N-terminal structures that are highly conserved among eukaryotic species ranging from *C. elegans* and *D. melanogaster* to humans^190,191^. Since the active sites of its exoribonuclease domain are in the N-terminus, XRN1 seems to have functional conservation among the species as well (Fig. 5)^191^. XRN1 is located primarily in the cytoplasm and acts as an essential degradation modulator for various RNAs, including mRNAs and miRNAs^190–192^. Because dysregulated gene expression is a critical driver of cancer, XRN1 may be closely associated with cancer progression by degrading specific RNAs that are important for maintaining normal cellular status. Several lines of evidence support XRN1 as a key player in cancers based on its intrinsic exoribonuclease activity^193,194^. However, the miRNA-modulating effects of XRN1 in various cancers have not been comprehensively reviewed yet. In this respect, several particular studies associated with its miRNA-regulatory effects in cancer and their related potential are considered in this section.

**Fig. 6:**
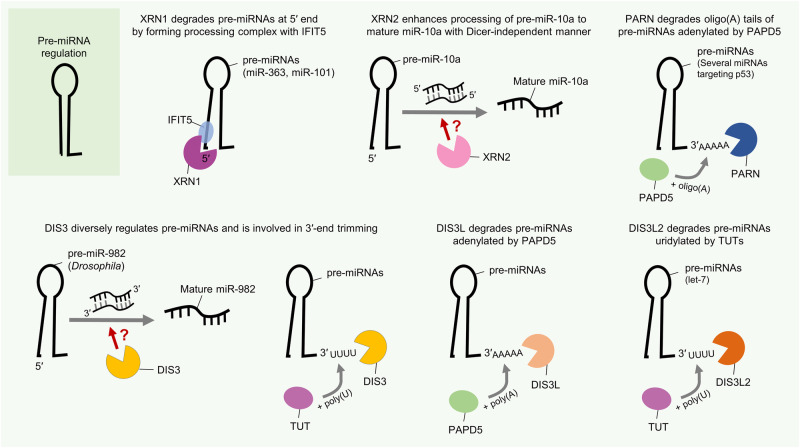
Exoribonuclease action in pre-miRNA regulation. The figure illustrates several examples of the mechanism of action of exoribonuclease-mediated pre-miRNA regulation in cancer. Various exoribonucleases have posttranscriptional mechanistic interactions with various pre-miRNAs. Consequently, exoribonuclease-triggered miRNA-dependent signaling pathways affect tumor progression. A detailed description of the mode of action is provided in the text.

XRN1 was shown to increase the expression of the androgen receptor by degrading miR-34a in prostate cancer^195,196^. Upregulation of the androgen receptor attenuates the expression of miR-204, which can target XRN1; consequently, XRN1 expression can be maintained at an aberrantly high level via this positive feedback loop. To exit this vicious cycle in prostate cancer treatment, XRN1 expression could be inhibited by either increasing miR-204 expression or directly targeting XRN1. Furthermore, XRN1 was suggested to play an important role in prostate cancer via diverse miRNA-regulatory functions. In another study, XRN1 was reported to be involved in the turnover of several tumor-suppressive miRNAs (miR-363, miR-101, and miR-128) at the precursor miRNA level by forming a processing complex with IFIT5, which is an IFNγ-induced EMT-stimulating gene (Fig. 6)^15^. IFIT5 can bind to pre-miRNAs by recognizing their 5′-end overhang structure, after then XRN1 is recruited to these IFIT5-bound pre-miRNAs. Consequently, XRN1 can ameliorate the effects of IFNγ-induced invasion in vitro and metastasis in vivo of prostate cancer.

Intriguingly, the miRNA decay function of XRN1 was also investigated for both extracellular miRNAs and endogenous miRNAs. XRN1 can promote the rapid decay of extracellular (ex-)miR-223-3p transferred from polymorphonuclear leukocytes (neutrophils) into cancer cells via extracellular vesicles^197^. The transferred ex-miR-223-3p inhibits the expression of its target FOXO1, thereby facilitating EMT and promoting invasive features in lung cancer cells. However, it was also found that the phenotypes induced by ex-miR-223-3p are only transiently maintained by rapid control of XRN1.

**Fig. 7:**
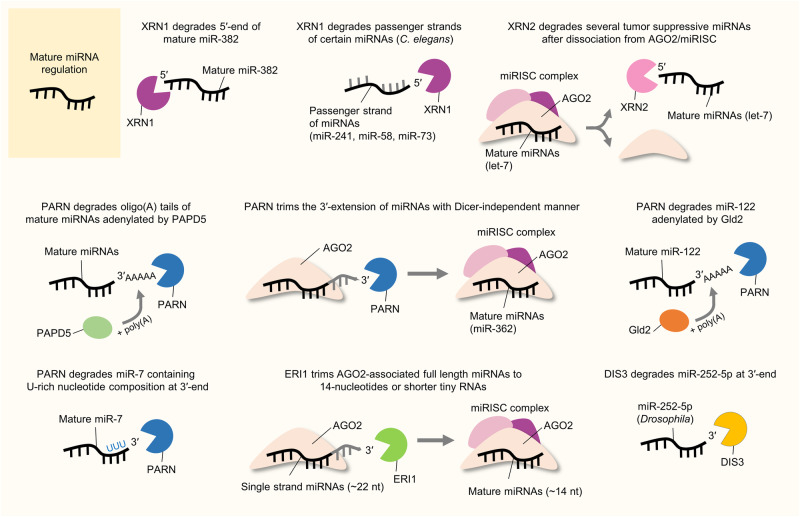
Exoribonuclease action in mature miRNA regulation. The figure illustrates several examples of the mechanism of action for exoribonuclease-mediated mature miRNA regulation in cancer. Various exoribonucleases have posttranscriptional mechanistic interactions with various naïve or posttranscriptionally modified mature miRNA species. Consequently, exoribonuclease-triggered miRNA maturation/trimming/degradation-dependent signaling pathways affect tumor progression. A detailed description of the mode of action is provided in the text.

In addition to these studies on cancer and cancer-related diseases, other potential mechanisms of action in miRNA regulation have been reported based on various model organisms ranging from humans (HEK293 cells) to *C. elegans* and *Drosophila*^192,198,199^. In human noncancerous cells, XRN1 but not XRN2 was shown to degrade miR-382 and affect its stabilization (Fig. 7)^192^. Although this finding was not verified in cancer studies, it could provide key preliminary data that could be applied to diverse human cancers, such as breast cancer and HCC, in which miR-382 has already been studied as a tumor-suppressive or an oncogenic miRNA^200,201^.

Other evidence from *C. elegans* and *Drosophila* studies also provided fundamental concepts that can be applied to human cells. For instance, depletion of XRN1, along with depletion of XRN2, can cause accumulation of the passenger strand of certain miRNAs (miR-241, miR-58, and miR-73) in *C. elegans* (Fig. 7)^198^. According to a *Drosophila* study, XRN1 (Pacman) can posttranscriptionally regulate miR-277-3p expression in wing imaginal discs via hypomorphic mutation of XRN1 (Pacman), suggesting that XRN1 can affect the maturation of miR-277-3p, not only its degradation^199^.

Taken together, this comprehensively accumulated evidence in various organisms supports the idea that XRN1 is a significant miRNA-modulating factor in human diseases, especially cancer. Furthermore, targeting its intrinsic RNA cleavage activity could be a potential therapeutic strategy for XRN1-dysregulated cancers by simultaneously regulating both its target mRNAs and miRNAs.

## 5′–3′ exoribonuclease XRN2

XRN2 is also one of the main 5′–3′ exoribonucleases, along with XRN1, that has highly homologous structures around the exonuclease domain within the N-terminal region (Fig. 5)^190,191^. Due to the similarity of its core region to that of XRN1, XRN2 can generally exhibit similar mechanisms of action^202^. However, it is known to be localized predominantly in the nucleus and nucleolus and has been shown to function in diverse aspects of RNA metabolism, such as transcriptional termination, telomere length regulation, and processing of rRNA and snoRNA^191^. In a similar context, XRN2 has also been investigated in cancers based on its intrinsic exoribonuclease activity. Like its paralog XRN1, XRN2 can process diverse RNA molecules, such as mRNAs, rRNAs, tRNAs, and miRNAs. This section comprehensively reviews several key findings about the miRNA-regulatory roles of XRN2 in cancer and its associated clinical potential.

Interestingly, XRN2 has been reported to be involved in not only miRNA degradation but also miRNA maturation in human cancer cells. Regarding its role in miRNA maturation, XRN2 was reported to induce EMT and metastatic behaviors in lung cancer cells by facilitating miR-10a maturation^16^. XRN2 can directly bind to pre-miR-10a and enhance its maturation in a Dicer-independent manner without affecting pri-miR-10a (Fig. 6). In addition to previous reports on the roles of miR-10 family members in cancer metastasis^203^, the authors found that miR-10a can target multiple genes involved in EMT, such as several HOX family members. Consequently, XRN2 was proposed to be related with poor patient prognosis and induce metastasis in lung cancer through EMT, which was at least partially promoted by miR-10a. These findings on the prometastatic miRNA-modulatory role of XRN2 also suggest the possibility of developing therapeutic strategies for XRN2-dysregulated metastatic cancers.

Regarding its role in miRNA degradation, XRN2 can degrade several tumor-suppressive miRNAs, including the let-7 family at the mature miRNA level (Fig. 7), without affecting the corresponding pri- or pre-miRNAs in lung cancer, HCC, and glioblastoma cells^204^. Consequently, XRN2 depletion leads to the accumulation of tumor-suppressive miRNAs, which can functionally target various oncogenes in cancer, and suppresses important cancer properties such as migration and invasion. Intriguingly, it was experimentally confirmed that XRN2 can degrade only tumor-suppressive miRNAs, not other oncogenic miRNAs, in lung cancer. These noticeable results could strongly support the practicality as a therapeutic target of blocking the tumorigenesis-driving factor XRN2 for cancer treatment. Moreover, the authors confirmed that XRN2 can target the mature let-7 miRNA after its dissociation from AGO2/miRISC via a putative unidentified miRNA-releasing factor (Fig. 7). This finding is consistent with a previous report demonstrating that mature miRNA activity is elicited via active processing by XRN2 in *C. elegans*. This study has shown that XRN2 is required for the degradation of mature miRNAs by promoting their dissociation from AGO proteins^205^.

Other research suggested that XRN2 can act as an amplified MYCN-induced mature miRNA-regulatory factor in neuroblastoma by actively degrading a subset of tumor-suppressive miRNAs, further contributing to cancer progression^206^. XRN2 expression was found to be induced by direct binding of MYCN to its promoter region in neuroblastoma cells, and two members of the let-7 family (let-7c and let-7g) were thus significantly upregulated in XRN2-depleted neuroblastoma cells. Although the biogenesis of let-7 miRNA family members was generally considered to be controlled by Lin28B in neuroblastoma, the aforementioned evidence indicated that XRN2 might be an important effector that modulates tumor-suppressive miRNAs in cancer.

Overall, the evidence indicates that XRN2 can selectively exert its functions to promote cancer progression and enhance malignant phenotypes by either promoting the maturation of oncogenic miRNAs or degrading tumor-suppressive miRNAs in cancer. However, the detailed mechanisms of its selective, preferential affinity remain to be elucidated. In addition, how XRN2 recognizes its target miRNAs involved in specific downstream signaling pathways in cancer should be further investigated.

## 3′–5′ exoribonuclease PARN

As one of the essential 3′–5′ exoribonucleases, poly(A)-specific ribonuclease (PARN) was initially identified as a pivotal 7-methylguanosine (m^7^G) cap-associated and poly(A) tail-modulating enzyme that plays an essential role in the translational control of mRNAs^207,208^. PARN was also shown to regulate cytoplasmic polyadenylation element-containing mRNAs by poly(A) shortening via competition with the noncanonical poly(A) polymerase Gld2 during *Xenopus* oocyte maturation^207,209^. Recent reports have demonstrated that PARN also contributes to the maturation and turnover of various noncoding RNAs, including miRNAs, via its intrinsic 3′–5′ exoribonuclease activity^209–211^. PARN is a 3′–5′ exoribonuclease that belongs to the DEDD 3′–5′ exonuclease domain superfamily and is structurally composed of an N-terminal R3H (arginine-three amino acid residues-histidine) motif within the 3′–5′ exonuclease domain, an RRM, and a C-terminal domain (CTD) (Fig. 5). Within the nuclease domain of PARN, three aspartate residues (D28, D292, and D382) and one glutamate (E30) residue were found to be essential for RNA catalysis; three of these residues (D28, E30, and D382) are also required for coordination of divalent metal ions, and substitution of any one of those amino acids abrogates the RNA catalytic activity of PARN^209^.

An earlier study demonstrated that PARN mediates the turnover of the key oncogenic miR-21. The results of exoribonuclease knockdown followed by small RNA sequencing suggested that PARN degrades miR-21, which is posttranscriptionally adenylated by the noncanonical poly(A) polymerase PAPD5 (Fig. 7). Knockdown of PAPD5 resulted in an increase in miR-21 expression, suggesting that PAPD5-mediated 3′-adenylation of miR-21 leads to its degradation^212^. Another report demonstrated that PARN modulates the biogenesis of the erythropoietic miRNA miR-451. The 3′-end of the AGO2-cleaved pre-miR-451 intermediate is then trimmed to the mature length via the 3′–5′ exoribonuclease activity of PARN (Fig. 7)^213^. PARN also specifically modulates miRNA stability. Hepatocyte-specific miR-122 is selectively stabilized by 3′-adenylation mediated by the cytoplasmic poly(A) polymerase Gld2, and it was reported that PARN is responsible for the deadenylation and destabilization of 3′-adenylated miR-122 (Fig. 7). RNA sequencing demonstrated that 3′-oligo-adenylated variants of miR-122 were enriched in Huh7 cells when PARN was depleted. Moreover, the steady-state level and stability of miR-122 were increased in PARN-knockdown cells^214^. Since miR-122 is known to function as a tumor-suppressive miRNA in HCC^37^, PARN may induce tumorigenesis in the liver and can be utilized as a therapeutic target.

Another interesting report showed the generation of the 3′-end of miRNAs by PARN in human cervical cancer cells. Lee and colleagues demonstrated that PARN trimmed the 3′ extensions of miRNAs that are derived from the genome or attached by terminal nucleotidyltransferases, thereby efficiently decreasing the length of these miRNAs. The authors suggested that PARN-mediated trimming processes may finalize miRNA maturation (Fig. 7)^210^. Shukla and colleagues demonstrated that the depletion of PARN in HeLa cells affects the levels of numerous miRNAs regulating p53 protein expression. They found that PARN removes oligo(A) tails from either mature miRNAs or pre-miRNAs that were added by the poly(A) polymerase PAPD5 (Fig. 6 and Fig. 7). Destabilization of these miRNAs caused by PARN-knockdown consequently increases p53 mRNA translation^215^.

A recent key study demonstrated that PARN activates EGFR-STAT3 signaling to exacerbate glioblastoma malignancy by degrading EGFR-targeting miR-7^19^. The authors confirmed that PARN promoted the self-renewal and proliferation of GSCs through positive modulation of EGFR expression via the negative regulation of EGFR-targeting miR-7 (Fig. 7). Then, increased EGFR expression led to the formation of a positive feedback loop to increase STAT3 activation^19^. The unbiased small RNA sequencing data also demonstrated that among the conserved miRNAs targeting EGFR mRNA, miR-7-5p was the most significantly upregulated after PARN-knockdown. More convincingly, the authors were able to monitor the U-rich nucleotide composition in the 3′-sequence of miR-7-5p, which is a relatively good substrate for PARN, based on a previous report that PARN efficiently degrades not only poly(A) but also poly(U) (Fig. 7)^216^.

Besides the miRNA-modulatory role of PARN, mutations in PARN also cause telomere diseases, including familial idiopathic pulmonary fibrosis and dyskeratosis congenita (DC)^217^. DC, a rare and complex inherited cancer predisposition syndrome, is caused by aberrant telomere biology. Moon and colleagues demonstrated that PARN is required for 3′-end maturation of telomerase RNA component (TERC). Deep sequencing of the TERC RNA 3′-terminus in somatic cells and induced pluripotent stem cells from DC patients with PARN mutations revealed that PARN is required for the removal of the posttranscriptionally acquired oligo(A) tail, which acts as a degradation signal^211^.

Although the cancer-related function of PARN is still under investigation, it has been reported that PARN is upregulated in gastric tumor tissues and gastric cancer cell lines^218^. In this study, PARN depletion was found to significantly inhibit the proliferation of gastric cancer cells and promote cell death. Depletion of PARN induced G_0_/G_1_ arrest in gastric cancer cells by upregulating the expression of p53 and p21^218^.

Collectively, these findings indicate that pharmacological and clinical approaches targeting single or multiple relevant components of the uninvestigated miRNA-modulatory network of PARN could be utilized to develop effective and precise cancer treatments to impede disease progression.

## 3′–5′ exoribonuclease ERI1

Enhanced RNAi 1 (ERI1; THEX1, 3′HEXO) is an evolutionarily conserved 3′–5′ exoribonuclease that is involved in the turnover of replication-dependent histone mRNAs in cooperation with stem‒loop binding protein (SLBP) and 3′-end trimming of 5.8 S rRNA^219^. Yeast ERI1 regulates the turnover of chromatin-associated siRNAs, whereas ERI1 in worms forms a complex with Dicer that generates specific classes of endogenous small interfering RNAs (endo-siRNAs)^219^.

ERI1 has a SAP domain with a conserved catalytic core that consists of four acidic amino acid residues (DEDD) that coordinate Mg^2+^ ions (Fig. 5). The enzymatic pocket accommodates only one to two nucleotides, which explains why 3′ single-stranded RNA overhangs are efficiently cleaved by ERI1, while double-stranded RNA is a poor substrate for ERI1. In mice, ERI1 regulates miRNA homeostasis in lymphocytes. Thomas and colleagues showed that ERI1 deficiency in mice repressed RNA interference and impaired maturation and Ly49 receptor expression in ERI1-null NK cells^220^.

During T-cell activation, extensive changes in the miRNA profile occur. Remarkably, upregulated miRNAs were shown to have a greater frequency of 3′-adenylation. Additionally, T-cell receptor stimulation was found to be followed by changes in the expression of RNA-modifying enzymes and RNA-degrading enzymes such as ERI1 and DIS3L2. This observation suggested that ERI1 may be involved in the maturation of a subset of miRNAs^221^. Sim and colleagues showed that several 3′–5′ exoribonucleases, including ERI1, are capable of trimming AGO-associated full-length miRNAs to 14-nt or shorter tiny RNAs (Fig. 7). These authors suggested that given the high Mn^2+^ concentrations in stressed and/or virus-infected cells, Mn^2+^-dependent exonucleases may have a role in remodeling gene silencing^222^.

Although the essential role of ERI1 in miRNA biogenesis has been commonly reported, there are very few reports of ERI1 being associated with human diseases, including cancer. Since ERI1 has enormous research potential, further investigations should be performed to identify the role of ERI1 in tumorigenesis.

## DIS3 3′–5′ exoribonuclease family

DIS3, which was initially identified in *Schizosaccharomyces pombe* mutants as *defective in sister chromatid disjoining*, is a highly conserved 3′–5′ exoribonuclease that belongs to the RNA II/RNB superfamily^223^. Controlled degradation of unnecessary RNA transcripts by the DIS3 family is essential for cell division and mitosis in eukaryotic cells. During the posttranscriptional regulation of gene expression, DIS3 family members are involved in the degradation of dispensable RNAs via their highly conserved 3′–5′ exoribonucleolytic activity^223,224^. The DIS3 family comprises three homologs, DIS3, DIS3L, and DIS3L2, in humans^225,226^. These processive 3′–5′ exoribonuclease family members share close structural similarities in their two cold-shock domains (CSD1 and CSD2), ribonuclease II (RNB) domain, and S1 domain but not in the N-terminal PilT (PIN) domain (Fig. 5). The central RNB catalytic domain is required to support RNA degradation^226^. The two tandem CSDs and the C-terminal S1 domain confer substrate RNA binding. The PIN domain is located in the N-terminus of DIS3 and DIS3L but not DIS3L2, which endows DIS3 and DIS3L with endonucleolytic activity and tethers DIS3 and/or DIS3L to the nine-subunit core (EXO9) in the RNA exosome complex^224^. DIS3L is also known to have defective endonuclease activity because of the alteration of two essential catalytic residues within its PIN domain. However, another homolog, DIS3L2, lacks the entirety of the PIN domain, suggesting that its role is independent of the RNA exosome. Compared with DIS3, DISL and DIS3L2 are mainly localized to the cytoplasm, where they contribute to the cytoplasmic RNA turnover pathway^226^. In vitro biochemical analyses also demonstrated that DIS3L2 possesses robust 3′–5′ exoribonucleolytic activity, degrading single- and double-stranded substrate RNAs^227^.

DIS3 and DIS3L are known mainly for their roles in the RNA exosome complex, and several reports have shown that these molecules are closely involved in miRNA biogenesis and cancer recurrence. During the formation of *Drosophila* wing imaginal discs, knockdown of DIS3 increases the expression of miR-252-5p, suggesting that DIS3 may function in the specific degradation of this miRNA during development (Fig. 7). Furthermore, the expression of mature miR-982-5p but not its precursor form was decreased in DIS3-knockdown discs, suggesting that DIS3 may be involved in precursor-to-mature miRNA processing (Fig. 6)^228^.

Moreover, Liu and colleagues reported that DIS3 cooperates with TUT4 and TUT7 to degrade pre-miRNAs (Fig. 6). DIS3 has distinct preferential activity toward pre-miRNAs, favoring those with >2 nt in the 3′ overhang. Knockdown of DIS3 does not seem to affect the level of mature miRNAs but does cause increases in the levels of several truncated pre-miRNAs, suggesting that DIS3 is involved in the quality control of pre-miRNAs^229^. In addition to directly modulating miRNA biogenesis, DIS3 promotes the maturation of the let-7 miRNA by degrading Lin28B mRNA in multiple myeloma (MM) cell lines. The reduction in mature let-7 caused by DIS3 depletion increases the translation of let-7 targets such as Myc and Ras, leading to accelerated tumorigenesis^230^.

Shukla and colleagues demonstrated that DIS3L and DIS3L2 are also critical 3′–5′ exoribonucleases that degrade miRNAs adenylated by PAPD5. They reported that in the absence of PARN, 3′-end adenylation leads to the recruitment of the cytoplasmic exoribonuclease DIS3L, which degrades miRNAs (Fig. 6)^215^. Similarly, two critical observations revealed that DIS3L2 is responsible for promoting pre-let-7 degradation in mouse embryonic stem cells^231,232^. DIS3L2 was found to preferentially degrade oligo-uridylated pre-let-7 (Fig. 6), and depletion of DIS3L2 resulted in the accumulation of oligo-uridylated pre-let-7, confirming that DIS3L2 is the key 3′–5′ exoribonuclease involved in pre-let-7 turnover via the Lin28-mediated cellular signaling pathway.

With respect to human diseases, DIS3 has been shown to be associated with various types of cancer, including colorectal cancer, melanoma, and three types of hematological malignancies. However, no obvious consensus has been reached on whether DIS3 functions as a tumor suppressor or as an oncogene. Specifically, in MM, an extremely serious hematological malignancy, DIS3 was identified as one of the most significantly mutated genes, which also included K-Ras, N-Ras, p53, BRAF, TRAF3, and FAM46C. DIS3 mutations and altered expression have been reported in approximately 10% of patients with MM^223,224,233^. More strikingly, deletions of another posttranscriptional modulator, FAM46C, a noncanonical poly(A) polymerase, have been observed in ~20% of MM patients^234^. These observations imply that posttranscriptional regulation of RNA metabolism mediated by DIS3 and its functional partner is critical for MM pathogenesis.

Another DIS3 family protein, DIS3L2, was recently reported to be closely related to the onset of Perlman syndrome, which is characterized by familial renal dysplasia, fetal gigantism, and multiple congenital anomalies. Perlman syndrome is inherited in an autosomal recessive manner and is closely associated with susceptibility to Wilms tumor^227^. DIS3L2 depletion was associated with mitotic abnormalities and altered expression of mitotic checkpoint proteins. DIS3L2 overexpression suppressed the growth of human cancer cell lines, and DIS3L2 knockdown increased cell growth. These observations suggest that DIS3L2 plays a critical role in RNA metabolism and is essential for the regulation of cell growth and division^227^. DIS3L2 is also reported to be overexpressed in CRC tissues and is associated with poor prognosis in CRC patients. Depletion of DIS3L2 impairs the viability, migration, and invasion of CRC cells by disrupting the mTOR signaling pathway. This observation suggests that DIS3L2 is required for sustaining CRC cell proliferation and the invasive behavior of dedifferentiated CRC cells^235^. Beyond the intrinsic 3′–5′ exoribonuclease activity of DIS3L2, aberrant DIS3L2 expression has also been reported to promote HCC progression via hnRNP U-mediated alternative splicing. Independent of its exoribonuclease activity, DIS3L2 directly interacts with hnRNP U through its CSDs, promoting the generation of an oncogenic splicing variant of Rac1b via exon 3b inclusion, which is known to stimulate cellular transformation and tumorigenesis. More importantly, the expression of both DIS3L2 and Rac1b is strongly correlated with HCC progression and patient survival^236^.

Taken together, the miRNA-regulatory functions of the DIS3 family members have been actively investigated, and their potential clinical importance has been continually reported. However, further investigations are required to elucidate the associations of their miRNA-regulatory roles with cancer progression. In the future, DIS3 family members could be applied in pharmacological and clinical targeted therapeutic approaches related to cancer treatment.

## The key players in HCV proliferation via the modulation of miR-122 and consequent development of HCC

Some RBPs and exoribonucleases described in this review have been shown to play a critical role in HCV proliferation via versatile crosstalk with the HCV RNA genome and/or miR-122, a liver-specific miRNA closely associated with HCV translation/replication (Table 3)^237^. Because HCV infection has been confirmed to cause hepatitis and ultimately contribute to HCC development^5,6^, approaches for targeting key related factors may be concurrently applied to control miR-122-associated HCV proliferation and utilized as therapeutic strategies for HCC.

**Table 3:** Summary of key RBPs and exoribonucleases associated with HCV proliferation via modulation of miR-122.

Type	Name	Role in HCV proliferation	Mechanisms of action associated with miR-122 regulation	Ref.
RBP	hnRNP E2	HCV translation (▲)	Competes with miR-122 for HCV RNA and functions in facilitating the translation of HCV RNA	^238^
	hnRNP K	HCV replication (▲)	Stabilizes and accumulates miR-122 by binding both miR-122 and HCV RNA based on the partially overlapping binding sites	^239^
	ELAVL1/HuR	HCV replication (▲)	Stabilizes miR-122 by physically associating with its 3′-end and positively affects miR-122 expression at the posttranscriptional level	^14,240^
Exoribonuclease	XRN1	HCV replication (▼)	Competes with miR-122 for HCV RNA by attacking and degrading 5′UTR of HCV RNA	^17,241,242^
	XRN2	HCV replication (▼)	Competes with miR-122 for HCV RNA by attacking the 5′UTR along with XRN1 and might have its own antiviral functions in the specific HCV genotypes	^18^

**Fig. 8:**
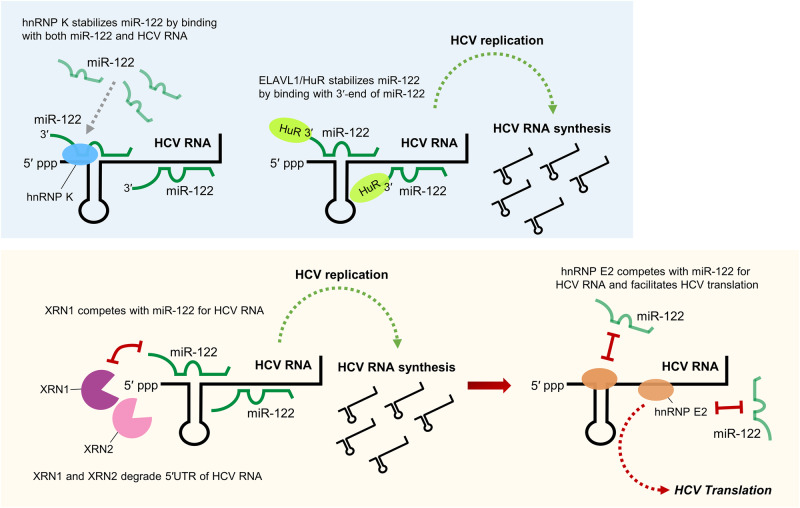
Effects of various RBPs and exoribonucleases on HCV genomic RNA and the HCV guardian miR-122. During HCV proliferation, hnRNP K may function as a key modulator of HCV RNA replication by recruiting intracellular miR-122 to HCV RNA. ELAVL1/HuR can physically bind to the 3′-end of miR-122 and positively influence the expression of miR-122. ELAVL1/HuR also significantly affects HCV proliferation through miR-122 regulation. XRN1 and XRN2 can compete with miR-122 for HCV RNA synthesis, whereas hnRNP E2 may compete with miR-122 for HCV RNA to facilitate HCV translation. A detailed description of the mode of action is provided in the text.

For instance, some hnRNPs were reported to function in HCV proliferation by modulating miR-122. HnRNP E2, also known as PCBP2, was found to compete with miR-122 in HCV-infected liver cancer cells (Fig. 8)^238^. Whereas miR-122 promotes HCV RNA replication, hnRNP E2 functions in the translation of HCV RNA (Fig. 8). Because miR-122-induced HCV RNA replication is required for hnRNP E2 to actively participate in viral protein translation, miR-122 promotes HCV RNA synthesis prior to active translation (Fig. 8). Intriguingly, the authors also showed that translation inhibition of HCV via depletion of hnRNP E2 subsequently prevented miR-122 from increasing HCV RNA synthesis^238^. Based on these results, it would be intriguing and plausible to impede HCV infection and consequent HCC progression through therapeutic interventions with selective hnRNP E2 inhibitors.

Moreover, hnRNP K was reported to be associated with both miR-122 and HCV RNA at partially overlapping binding sites^239^. Notably, miR-122 can be stabilized and accumulate upon binding to hnRNP K (Fig. 8). Based on these results and those of previous studies, hnRNP K may function as a key modulator of HCV replication by recruiting intracellular miR-122 to HCV RNA (Fig. 8).

Intriguingly, Seo and colleagues showed that ELAVL1/HuR is physically associated with the 3′-end of miR-122 and positively influences the expression of miR-122 at the posttranscriptional level (Fig. 8)^14,240^. Furthermore, ELAVL1/HuR significantly affects HCV proliferation through direct miR-122 regulation (Fig. 8)^14^. While the competitive effect of ELAVL1/HuR and miR-122 on CAT-1 mRNA translation in HCC was described in a previous section^76^, the authors essentially demonstrated the direct effect of the interplay between ELAVL1/HuR and miR-122 on HCV proliferation. Indeed, the authors proposed that ELAVL1/HuR may exert diverse effects during each step of the RNA life cycle as a complex with miR-122, depending on the target RNA and cellular context.

Furthermore, it was reported that XRN1 can compete with miR-122 for HCV RNA synthesis by attacking the 5′UTR of HCV RNA, whereas HCV RNA can be protected and stabilized from 5′–3′ exoribonuclease-mediated decay by forming a protective complex with miR-122 at the 5′-end of the viral RNA (Fig. 8)^17,241,242^. These observations suggest that HCV RNA replication may be attributed to the competition between XRN1 and miR-122 for HCV RNA. Interestingly, exogenous miR-122 supplementation did not affect the HCV RNA abundance under the conditions of XRN1 depletion. This result indicated that the effects of XRN1 depletion and miR-122 supplementation on HCV RNA protection are mostly redundant. However, Li and colleagues confirmed that the decay-promoting effect of XRN1 can be observed in diverse HCV strains, suggesting that XRN1 is a primary 5′-end degradation factor of HCV RNA in multiple virus strains^241^. Therefore, activation of XRN1 could be utilized to develop selective therapeutic strategies targeting most HCV genotypes.

Similar to XRN1, XRN2 can decrease the abundance of HCV RNA by degrading its 5′UTR along with XRN1 (Fig. 8)^18^. Whereas miR-122 supplementation did not affect the HCV RNA abundance under conditions of XRN1 depletion, XRN2 depletion exerted an additive effect on HCV RNA protection in combination with miR-122 supplementation^17,18^. Based on these results, XRN2 may have its own antiviral functions that differ from those of XRN1, except for competition with miR-122 for HCV RNA, in specific HCV genotypes. However, unlike XRN1, XRN2 has been reported to be exclusively effective in a few HCV strains^241^. Therefore, combining miR-122 inhibition and XRN2 activation should be considered when developing more effective therapies for HCC-causing HCV infections based on the viral genotype.

## Therapeutic strategies for targeting RBPs and exoribonucleases modulating miRNAs in cancer

As described in the above sections, miRNA-modulating RBPs and exoribonucleases play indispensable roles in various steps of tumorigenesis. However, relatively little attention has been given to strategies for targeting and inhibiting these factors. To date, these molecules have been considered ‘hard to be druggable’ compared to other key signaling molecules, such as receptor tyrosine kinases (RTKs). Recently, the important functional roles of these posttranscriptional molecular networks in cancer have continually emerged. In this section, we describe the strategies for inhibiting the aforementioned key players by using antisense oligonucleotides (ASOs) and small-molecule inhibitors for direct and/or indirect targeting of miRNAs, miRNA-modulating RBPs and exoribonucleases.

As therapeutic strategies involving oligonucleotide-based drugs, ASOs and miRNA mimics have shown potential in both bench studies and clinical trials. In pancreatic cancer, HCC, myeloma, and glioblastoma, which exhibit elevated expression of miR-21, ASO-miR-21 was shown to inhibit cell proliferation. As miRNA inhibitors, the HCV-LNA therapeutics Miravirsen and RG-101 have been shown to effectively target miR-122 in HCV infections^243,244^. Moreover, the results obtained with MRX34, a miR-34 mimic oligomer, provided proof of concept for miRNA-based cancer therapies in the clinic^245^.

Small-molecules are by far the most commonly used compounds to target RBP function in cancers and other human diseases. Several of these molecules have shown promising anticancer activity in preclinical studies, and some of them are currently being evaluated in clinical trials. Small molecules can be used to inhibit miRNA-modulating RBPs and exoribonucleases function in one of the following ways: 1) by binding to the RBD and subsequently impeding RBP-miRNA interactions; 2) by inhibiting the enzymatic activity of RBPs and exoribonucleases toward their target miRNAs; and 3) by inhibiting the transcription and posttranscriptional signaling pathway of the target miRNA-modulating RBPs and exoribonucleases.

The abovementioned RBP ELAVL1/HuR has been identified as a promising target for RBP-based therapeutics. The small-molecule inhibitor MS-444, which was discovered by Meisner and colleagues, was used to inhibit the binding of ELAVL1/HuR by preventing its dimerization^246^. Other small-molecule inhibitors, such as the microtubule inhibitor MPT0B098 and abemaciclib, were able to inhibit ELAVL1/HuR activity, resulting in reduced tumor growth^247^. Recently, rigosertib, which is an anticancer drug candidate currently in clinical trials, has also been shown to effectively decrease the levels of both ELAVL1/HuR and miR-122, which was reflected in the significant reduction in HCV proliferation^14^. This suggests that targeting RBPs could be an effective method for indirectly targeting miRNAs to prevent disease progression.

The possibility of inhibiting other RBPs, such as Lin28, with small-molecule inhibitors has also been explored. Lekka and colleagues reported that the small-molecule C1632 could inhibit Lin28 binding to let-7 precursors^248^. In addition, Wang and colleagues reported that various selective Lin28 inhibitors, such as TPEN and LI71, can suppress Lin28 by targeting various individual domains of Lin28^249^.

DEAD-box helicases, which are overexpressed in many cancer types, have also been investigated as RBP targets for small-molecule inhibitors. After the screening of potential inhibitor, RK-33, which was designed by Bol and colleagues, was demonstrated to effectively induce loss of function of DDX3, which caused cell cycle arrest, apoptosis, and radiosensitization^250^. Yin and colleagues also demonstrated that the small-molecule inhibitor ivermectin, a macrocyclic lactone, could suppress the proliferation of both GSCs and glioblastoma cells through the inhibition of DDX23^188^. Furthermore, GW4064, another small-molecule inhibitor, was found to be a potent inhibitor of DDX23 in the context of miR-21 inhibition in NSCLC and pancreatic cancer cells^251^.

In regard to the strategy of inhibiting exoribonucleases with small-molecule inhibitors, the related reports are relatively deficient; however, there are several reports related to PARN. Yin and colleagues recently reported that the small-molecule STAT3 inhibitors stattic and niclosamide could inhibit STAT3-mediated PARN expression and consequently affect miR-7 turnover^19^. In addition, small-molecule inhibitors of PARN, such as GNF-7, were identified through computational-based docking analysis and high-throughput screening and were demonstrated to reduce the levels of specific RNAs through PARN inhibition^252^.

Thus, by looking beyond conventional miRNA modulators to various other RBPs and exoribonucleases involved in miRNA maintenance and turnover, diverse therapeutic strategies can be developed. While many existing strategies involve small-molecule-based inhibition, the emerging oligonucleotide-based therapeutics may soon be applied to the targeting of RBPs and exoribonucleases, expanding the current scope of RNA therapeutics.

## Concluding remarks and future perspectives

This review comprehensively discusses RBPs and exoribonucleases as miRNA regulators in various diseases, including cancers and a representative cancer-causing viral disease (HCV). To date, the aspects of molecular dysregulation caused by miRNAs in cancers have been actively reported and are relatively well understood. However, the functional roles of the molecular factors modulating miRNAs in cancers have not been sufficiently reviewed yet. In that sense, this review will be helpful for understanding both the well-established existing roles and recently reported new evidence on the miRNA-regulatory mechanisms of RBPs and exoribonucleases.

The effects of these factors on miRNA regulation are quite different across cancers depending on the type of miRNA and the cellular context. In particular, several RBPs (e.g., ELAVL1/HuR) have been shown to have dual functions in miRNA regulation; i.e., both competitive and cooperative roles. Others (e.g., KSRP) have been reported to consequently exhibit dual effects on cancer progression via each miRNA-regulatory mechanism; i.e., promotive and inhibitory effects. Exoribonucleases have also been shown to be involved not only in miRNA degradation but also in miRNA maturation. Therefore, prior to evaluating the potential effects of these miRNA regulators in terms of therapy, a thorough understanding of the omnidirectional miRNA-regulatory mechanisms and dual functions of various miRNAs is needed.

Based on this complexity, targeting these miRNA-modulating factors might be more difficult than targeting historically evaluated signaling molecules. However, continuous investigations of the molecular interplay between miRNAs and their intracellular regulatory factors have made extensive progress toward the development of new therapeutic strategies, such as ASO drugs and small-molecule inhibitors. Currently, steadily accumulated evidence regarding various RBPs and exoribonucleases has not only proven their potential as therapeutic targets but also raised the possibility of developing new types of clinical therapeutics for cancer treatment. In this regard, this comprehensive overview is helpful to take a step forward in understanding and realizing the therapeutic potential of RBPs and exoribonucleases for cancer treatment.

However, several miRNA regulators, such as ERI1 and DIS3, have not yet been fully investigated for their roles in regulating miRNAs in cancers. Since there are many fields in which new information about these factors can be discovered, further investigations will most likely identify new roles of these factors in miRNA regulation during tumorigenesis. In addition, the detailed mechanisms of these miRNA regulators in cancers have not been fully elucidated. Thus, how RBPs and exoribonucleases can regulate miRNAs in the specific cellular environment of cancer should also be further investigated.
